# The Secretome Deregulations in a Rat Model of Endotoxemic Shock

**DOI:** 10.1155/2021/6650464

**Published:** 2021-07-24

**Authors:** A. Blangy-Letheule, A. Persello, S. Michelland, V. Cunin, F. Souab, V. Aillerie, J. Dhot, A. Erraud, J. Montnach, M. Seve, S. Bourgoin-Voillard, B. Rozec, M. De Waard, B. Lauzier

**Affiliations:** ^1^Université de Nantes, CHU Nantes, CNRS, Inserm, L'Institut du Thorax, F-44000 Nantes, France; ^2^InFlectis BioScience, Nantes, France; ^3^Univ. Grenoble Alpes, LBFA and BEeSy, Inserm, U1055, PROMETHEE Proteomic Platform, Saint-Martin-d'Hères, France; ^4^CHU Grenoble Alpes, Institut de Biologie et de Pathologie, PROMETHEE Proteomic Platform, Grenoble, France; ^5^University of Grenoble Alpes, CNRS, Grenoble INP, TIMC, PROMETHEE Proteomic Platform, 38000 Grenoble, France; ^6^University of Paris-Est Créteil (UPEC), Inserm U955, Equipe 21, UMR_S955, APHP, Hôpital H. Mondor-A. Chenevier, Centre d'Investigation Clinique Biothérapie, Créteil, France; ^7^LabEx Ion Channels, Science and Therapeutics, F-06560 Valbonne, France

## Abstract

**Introduction:**

Septic shock is a systemic inflammatory response syndrome associated with organ failures. Earlier clinical diagnosis would be of benefit to a decrease in the mortality rate. However, there is currently a lack of predictive biomarkers. The secretome is the set of proteins secreted by a cell, tissue, or organism at a given time and under certain conditions. The plasma secretome is easily accessible from biological fluids and represents a good opportunity to discover new biomarkers that can be studied with nontargeted “omic” strategies.

**Aims:**

To identify relevant deregulated proteins (DEP) in the secretome of a rat endotoxemic shock model.

**Methods:**

Endotoxemic shock was induced in rats by intravenous injection of lipopolysaccharides (LPS, *S. enterica typhi*, 0.5 mg/kg) and compared to controls (Ringer Lactate, *iv*). Under isoflurane anesthesia, carotid cannulation allowed mean arterial blood pressure (MAP) and heart rate (HR) monitoring and blood sampling at different time points (T0 and T50 or T0 and T90, with EDTA and protease inhibitor). Samples were prepared for large-scale tandem mass spectrometry (MS-MS) based on a label-free quantification to allow identification of the proteins deregulated upon endotoxemic conditions. A Gene Ontology (GO) analysis defined several clusters of biological processes (BP) in which the DEP are involved.

**Results:**

Ninety minutes after shock induction, the LPS group presents a reduction in MAP (-45%, *p* < 0.05) and increased lactate levels (+27.5%, *p* < 0.05) compared to the control group. Proteomic analyses revealed 10 and 33 DEP in the LPS group, respectively, at 50 and 90 minutes after LPS injection. At these time points, GO-BP showed alterations in pathways involved in oxidative stress response and coagulation.

**Conclusion:**

This study proposes an approach to identify relevant DEP in septic shock and brings new insights into the understanding of the secretome adaptations upon sepsis.

## 1. Introduction

Septic shock is responsible for one death every 2.8 seconds worldwide [1]. In 50% of cases, septic shock causes myocardial dysfunction resulting in over 60% excess global mortality [2, 3]. The dynamics of septic shock consist of a compensation phase followed by decompensation. The kinetics of the different phases of the pathology varies according to the patient, resulting in a variation in the kinetics of organ dysfunctions from one patient to another. Limiting the development of cardiovascular dysfunctions and optimizing care would increase the patient's chances of survival. Septic shock is multifactorial and presents a large heterogeneity between patients. This point explains the variable kinetics of pathology development and makes it difficult to investigate causal mechanisms [4]. The early use of sensitive and specific biomarkers of septic shock clinical evolution would facilitate rapid diagnosis and early management of patients. Such biomarkers must be easily accessible and quickly analyzable for clinical use. Unfortunately, due to the complexity of the pathology, such biomarkers are not currently available for clinicians [5]. The use of animal models allows investigators to considerably limit the heterogeneity between individuals, while, at the same time, improving access to samples before shock initiation and throughout the progression of the pathology. The *in vivo* administration of lipopolysaccharides (LPS), which triggers endotoxemic shock, is one of the most common and simple ways to model shock in animals [6].

In this context, the study of the secretome, defined as the set of proteins secreted by a cell, tissue, or organism at a given time and under certain conditions, is a relevant approach [7]. Changes in plasmatic secretome composition may reflect a pathological state [8]. Indeed, injection of plasma from patients diagnosed with septic shock in a healthy mouse constituted the first proof of concept in 2011 that the secretome could provide information on this pathology. This approach demonstrated the presence of circulating blood factors causing the physiopathology of septic shock [9]. The proteins that are deregulated in the secretome of septic shock patients could potentially be used as biomarkers of the septic shock prognosis. The objective of this study is to identify early biomarkers of septic shock by studying sepsis prior to and at the acute stage of the decompensation phase. For this purpose, the plasma secretome in a rat model of endotoxemic shock was studied at two time points using a large-scale nontargeted mass spectrometry (MS) approach. The design of this study made it possible to follow each subject before shock induction and then at two important time points of the development of the pathology. Through this approach, we identified deregulated proteins (DEP) of the acute stage of shock that is associated with the deregulation of numerous biological processes such as coagulation or the response to oxidative stress.

## 2. Methods

### 2.1. Animals

Twelve-week-old male Wistar rats were housed under standard conditions of temperature (21-24°C), humidity (40-60%), and a 12-hour light/dark cycle. Food and water were available *ad libitum*. Experiments were approved by the ethics committee in charge of animal experimentation, the committee of the Pays de la Loire (12760-2017121810244298), and were performed in accordance with the French law on animal welfare, EU Directive 2010/63/EU for animal experiments, and the National Institutes of Health (NIH).

### 2.2. Endotoxemic Rat Model

**Figure 1:**
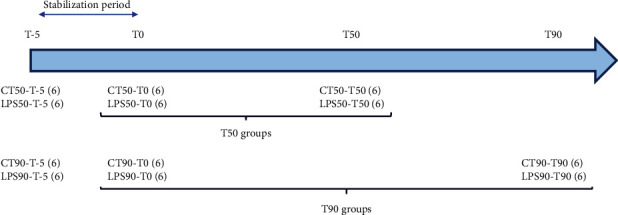
Group repartition for the 12-week-old Wistar rats. The period between T-5 and T0 corresponds to stabilization. T0 is the control before solvent or LPS injection and therefore concerns the same rats 50 or 90 minutes after injection. Each group consists of 6 rats (6).

Endotoxemia was induced by intravenous injection of 0.05 mg·kg^−1^ of purified LPS obtained from *Salmonella enterica* serotype *typhimurium* (batch 078M4021V, Sigma, St. Louis, USA) suspended in Ringer Lactate (RL, B. Braun, France) as previously described [10]. Rats were randomly distributed into four groups: the control group, having received an injection of RL, and LPS groups followed during 50 minutes (CT50 and LPS50, respectively) or during 90 minutes (CT90 and LPS90, respectively), with 6 animals per group. In each group, animals are their own control meaning that each group comprised a T0 time point and either a T50 or a T90 time point (Figure 1).

### 2.3. Monitoring

Animals were anesthetized with 2% volume of isoflurane and 0.6 L·min^−1^ O_2_ to limit hemodynamic effects of anesthesia. Arterial blood pressure measurements were performed through the left carotid artery to calculate the mean arterial blood pressure (MAP) and heart rate (HR). Briefly, the right carotid artery was isolated and ligated at the distal end, and a PE-50 catheter containing Ringer Lactate was inserted. Pressure signal and HR were continuously recorded, displayed, and stored by the IOX® software (EMKA Technologies, Paris, France).

### 2.4. Blood Analyses and Plasma Preparation

A volume of 500 *μ*L or 5 mL of arterial blood was, respectively, collected at T0 and at the terminal endpoint (50 or 90 minutes) through the carotid catheter. At T0, vascular filling with RL was performed to compensate for the volume collected. Blood samples were placed in 2 mL EDTA tubes (Sarstedt AG & Co. KG, Numbrecht, Germany) containing protease inhibitors (Complete ULTRA Tablet, Mini, Protease Inhibitor Cocktail, MERCK laboratory). Lactate concentration was measured from 10 *μ*L of venous blood using Nova StatStrip (Nova Biomedical, Rungis, France). For terminal sampling, blood gases, electrolytes, and metabolites (BGEM) were measured from 90 *μ*L of arterial blood using a BGEM card (Siemens Healthcare™, Ottawa, Canada) and analyzed by the ePOC analyzer (Siemens Healthcare™, Ottawa, Canada). Plasma was obtained by blood centrifugation (10 minutes, 1,300 g) at room temperature (RT) and frozen in liquid nitrogen. Thereafter, samples were stored at -80°C.

### 2.5. Sample Preparation for Label-Free Mass Spectrometry Analyses

Plasmatic protein concentration was determined by colorimetry using the BCA method (BiCinchoninic acid Assay, Thermo Fisher Scientific, Walt-man, Massachusetts, United States). The absorbance was measured at 560 nm using a Varioskan reader (Thermo Fisher Scientific, Walt-man, Massachusetts, USA). Plasmatic samples were enriched for low abundance proteins using ProteoMiner™ kits (Bio-Rad, Hercules, USA), in accordance with the manufacturer's instructions. The plasma volume loaded on the ProteoMiner™ column contained 10 mg of total proteins per column. In order to remove salts, the eluate was loaded in 2 kDa dialysis cassette (Slide-A-Lyzer, Thermo Fisher Scientific, United States) in a 50 mM ammonium bicarbonate dialysis buffer (pH = 7.7 ± 0.1, batch BCBZ3540, Sigma-Aldrich, St. Louis, USA). The concentration of proteins present in the dialysate was determined as previously described. Aliquots were finally prepared in order to obtain 25 *μ*g of proteins and stored at -80°C prior to the mass spectrometry analysis. Prior to mass spectrometry, 25 *μ*g of proteins of each sample was reduced with Dithiothreitol (DTT, Sigma-Aldrich) at 56°C for 1 h, alkylated with iodoacetamide (Sigma-Aldrich) at 37°C for 30 min in the dark, and digested at 37°C overnight simultaneously with both trypsin and Lys-C enzymes (Trypsin/Lys-C Mix, Mass Spec Grade, Promega, France) using an enzyme/protein ratio of 3 : 100. Each sample was spiked with 700 fmol of enolase digest from *Saccharomyces cerevisiae* (Waters, Milford, United Kingdom). Resulting peptides were desalted with C18 spin columns with a peptide binding capacity of 30 *μ*g (Pierce C18 Spin Columns, Thermo Fisher Scientific, Walt-man, Massachusetts, United States). Two micrograms of each sample was mixed to prepare a Quality Control (QC) sample. Samples were dried by using a speed vacuum (Eppendorf) and stored at -20°C until label-free high-mass resolution/accuracy mass spectrometry analyses.

### 2.6. Liquid Chromatography and High-Resolution Accurate-Mass (HRAM) Mass Spectrometry Analyses

Protein quantitation was performed according to a label-free quantitative proteomic approach based on a high-resolution accurate-mass (HRAM) mass spectrometry analysis. For this purpose, samples (including QC to verify the stability of the signal) were analyzed by an UHPLC-MS (HRAM) system, a Vanquish Flex Binary UHPLC system (Thermo Fisher Scientific, France) coupled with a Q Exactive Plus mass spectrometer (Thermo Fisher Scientific, France) equipped with an electrospray ionization source operating in a positive mode and an Orbitrap mass analyzer. Peptides resuspended in buffer A (2% ACN, 0.1% FA) were loaded on the Vanquish UPLC equipped with a Viper Fingertight Fittings Column Protection (Viper Inline Filter, Titanium, av. Frit presize 0.5 *μ*m) and a C18 column (ACCL RSLC120 C18, 2.2 *μ*m, 120 Å, 2.1 mm × 250 mm). A binary gradient of 120 min of buffer A (99.9% H_2_O, 0.1% FA) and buffer B (99.9% ACN, 0.1% FA) at a flow rate of 0.4 mL/min and at 60°C set up as follows: 0–2 min, 1-3% B; 2–55 min, 3–15% B; 55–95 min, 15–38% B; 95–105 min, 38–95% B; 105–109 min, 95% B; 109–110 min, 95–1% B; and 110–120 min, 1% B was applied to separate peptides. MS analyses were performed upon a full MS mode with a resolution of 70,000, a maximum IT of 17 ms within a scan range of 200-2,000 *m*/*z*, and a lock-mass ion at *m*/*z* 445.120024 of polycyclodimethylsiloxane from the ambient air. The external calibration was done with a CalMix calibrant (Pierce, Thermo Fisher Scientific, Walt-man, Massachusetts, United States) in positive mode by considering ±5 ppm of mass tolerance. MS/MS analyses were performed by using a data-dependent acquisition based on HCD (higher-energy collisional dissociation) activation mode with an isolation window of 4 u, a resolution of 17,500, an automatic gain control target of 2.10^5^, and a maximum IT of 200 ms on the 10 most intense ions. A dynamic exclusion of 10 s was applied. Each sample was analyzed in duplicate. The data were acquired with Thermo Scientific™ XCalibur 2017 v.4.1.31.9. software.

### 2.7. Data Analyses

The identification of proteins was achieved thanks to PEAKS®X Studio 10.0 software [11] and the UniProtKB database of *Rattus norvegicus* (UP000002494, Release 2020_01) uploaded with the enolase protein from *Saccharomyces cerevisiae* (P00924). The following criteria were applied for the protein identification: a fixed modification of carbamidomethylation, variable modifications of oxidation (HW), oxidation (M), acetylation (protein N-term), 2 missed cleavages, at least 2 unique peptides, a tolerance MS of 5 ppm, a tolerance MS/MS of 0.01 Da, a peptide FDR of 1%, and a protein-10Log*p* of 20. The identification was done by using PEAKS DE Novo, PEAKS database, and SPIDER tools. The label-free protein quantitation was done by considering a retention time shift tolerance of 3 min and the enolase 1 protein from *Saccharomyces cerevisiae* (P00924) as an internal standard for the signal normalization. The quantity of proteins after LPS injection was compared in each group of six biological replicates with paired controls as detailed in the previous section. LPS and RL administration in Wistar rats allowed the study of (i) changes in the secretome 50 min postinjection (group CT50-T0 *vs.* CT50-T50 and group LPS50-T0 *vs.* LPS50-T50) and (ii) secretome adaptations 90 min LPS postinjection (group CT90-T0 *vs.* group CT90-T90 and group LPS90-T0 *vs.* LPS90-T90). A total of 48 samples, with technical duplicates for each of them, were therefore analyzed. The protein quantitation was performed only on proteins in which at least two peptides were quantified within a chromatography retention time range between 0 and 120 min with a score quality ≥ 6. This approach led to the identification of 401 proteins in the T50 group and 514 proteins in the T90 group.

**Figure 2:**
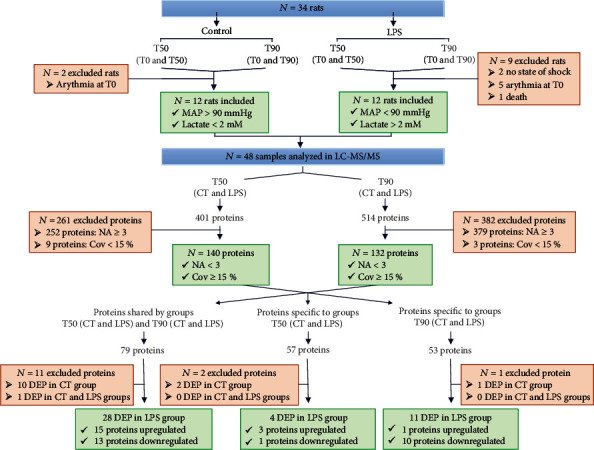
Flow chart of inclusion and exclusion procedure used to obtain a list of deregulated protein. The animals were divided into 4 groups (CT50, LPS50, CT90, and LPS90). Physiological parameters made it possible to include 24 rats in the study. For each rat, 2 time points were studied (T0 and T50 or T0 and T90) and were analyzed by mass spectrometry (*N* = 48 samples), and proteomic results were analyzed by RStudio software. Proteins with a maximum of 2 missing data (NA) per sample and a coverage (Cov) greater than 15% were selected. A total of 140 proteins in group T50 and 132 proteins in group T90 were included. The deregulated proteins (DEP) present in the controls were excluded from the study.

Proteins with missing values (indicated by the NA symbol) in at least 3 samples per group and less than 15% of coverage were withdrawn (Figure 2). The quantified proteins were considered deregulated only if they have a fold change higher than 2 (Log_2_FC ≥ 1) or lower than -2 (Log_2_FC ≤ −1) in at least 4 samples over the 6 in each group. 14 deregulated proteins in the control groups were excluded from the study (Figure 2).

The clustering of the DEP was analyzed using the STRING protein database for Gene Ontology (GO) analyses. The results of the analyses include biological process (BP) and cellular component (CC).

### 2.8. Western Blot Analyses

Western blotting experiments were performed on plasma samples using an antibody directed against Gpx3 (13647-1-AP, Manchester, United Kingdom, Proteintech). Briefly, proteins were quantified using a BCA protein assay kit. 50 mg of each sample was separated on an SDS-PAGE gel and transferred to a nitrocellulose membrane. The membranes were blocked with 5% milk in TBS 1x-Tween 0.5x (TBS-T) and then incubated with primary antibody (Gpx3, 1 : 400) overnight at 4°C. After 4 washes with TBS-T, the membranes were incubated with an HRP-conjugated secondary antibody (anti-rabbit, 1 : 10 000, sc-2054, Santa Cruz Biotechnology). Analyses were performed using Image Lab software (Bio-Rad, California, United States). A ratio to the stain-free intensity was calculated.

### 2.9. Statistical Analyses

Hemodynamic results were expressed as the mean ± SEM of *N* different rats. For hemodynamic parameters, lactatemia, and creatininemia, data were analyzed by a two-way ANOVA test with Bonferroni post hoc test.

Analyses of Western blots were expressed in relation to the average of the protein quantification (stain-free) and then reduced to the average of the control samples (CT50-T0, CT90-T0, LPS-T0, and LPS-T90). Data were analyzed with a two-way-ANOVA test with repeated measures and a Bonferroni post hoc test.

A value of *p* < 0.05 was considered significant. All statistical calculations and graphs (except those performed with R software) were performed using GraphPad PRISM 8 software (8.4.2 version).

### 2.10. Data Availability

The proteomic data were deposited to the ProteomeXchange Consortium with the MassIVE identifier MSV000087803 (http://massive.ucsd.edu) and ProteomeXchange identifier PXD027255 (http://www.proteomexchange.org).

## 3. Results

### 3.1. Animal Model

#### 3.1.1. Effect of Endotoxemic Shock on Hemodynamic Parameters

**Figure 3:**
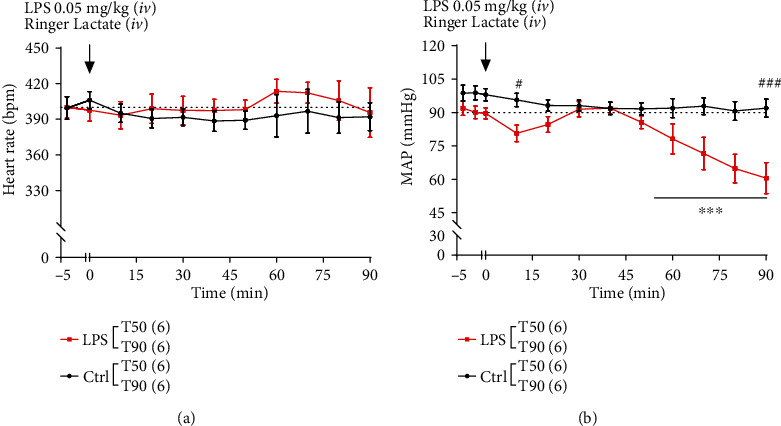
Effect of lipopolysaccharide (LPS) administration on heart rate (HR) and mean arterial blood pressure (MAP). Black and red squares represent the control and LPS rats, respectively. The dotted line represents the threshold considered physiological. The T50 and T90 groups are pooled for both control and LPS conditions (*n* = 12 in the control group and *n* = 12 in the LPS group). Only the T90 group is monitored after 50 minutes (*n* = 6 for the T90 control group and *n* = 6 for the T90 LPS group). Values represent the mean ± SEM of 6 rats per group. (a) Heart rate and (b) MAP were evaluated during 50 or 90 min after LPS injection and were expressed in beats per min (bpm) and mmHg, respectively. The symbol # represents a comparison at a given time between the CT and LPS groups, and the symbol ∗ represents a comparison over time for the LPS group. ^#^*p* < 0.01, ^###^*p* < 0.001, and ^∗∗∗^*p* < 0.001 two-way ANOVA test with Bonferroni post hoc test.

Continuous hemodynamic monitoring showed no significant changes neither in HR nor in MAP during the 5 min of stabilization (from T-5 to T0). MAP and HR remained stable in the control group at 90.0 ± 10.0 mmHg and 390.0 ± 10.0 bpm during the whole procedure. Injection of LPS (T0) induce any modification in HR (400.0 ± 10.0 bpm) (Figure 3(a)), whereas it was followed by an early decrease in MAP (Ctrl: 95.6 ± 2.9 mmHg *vs.* LPS: 80.6 ± 3.7 mmHg, *p* < 0.05, Figure 3(b)) with a return to basal values after 10 min. From 50 min after LPS injection to the end of the procedure, MAP decreased significantly (from 91.4 ± 3.4 mmHg at 50 min to 60.5 mmHg ± 6.9 mmHg at 90 min, *p* < 0.001, Figure 3(b)).

#### 3.1.2. Effect of Endotoxemic Shock on Plasmatic Biomarkers

**Figure 4:**
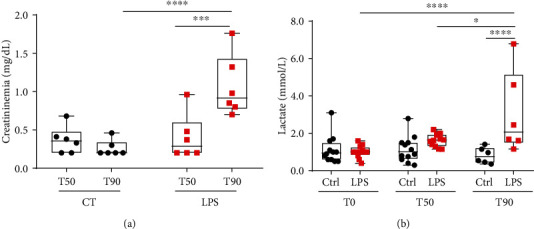
Variation of creatininemia (mg/dL) and lactatemia (mmol/L) throughout the development of endotoxemic shock. Black circles and red squares represent the control and LPS rats, respectively. (a) Arterial creatinine was measured at 50 or 90 minutes after shock induction; (b) venous lactates were measured at 0 and 50 or 0 and 90 min. Box & whiskers represent measures. (a, b) Were analyzed with a two-way ANOVA test with Bonferroni post hoc test. ^∗^*p* < 0.5, ^∗∗∗^*p* < 0.001, and ^∗∗∗∗^*p* < 0.0001.

Arterial creatinine and venous lactate concentrations were measured in the Ctrl and LPS groups at T50 and T90 and T0, T50, and T90, respectively (Figure 4). No change in creatininemia was observed at T50 between the Ctrl and LPS groups. However, creatininemia in the LPS T90 groups increases significantly when compared to time-matched Ctrl (Ctrl T90: 0.26 ± 0.04 mg/dL; LPS T90: 1.07 ± 0.16 mg/dL, *p* < 0.01, Figure 4(a)) indicating an alteration in renal function. Lactatemia remained in the normal range in Ctrl throughout the protocol while significantly increasing in the LPS T90 group (LPS T0: 1.02 ± 0.09 mmol/L *vs.* LPS T90: 3.04 ± 0.90 mmol/L, *p* < 0.001; LPS T50: 1.63 ± 0.10 mmol/L *vs.* LPS T90: 3.04 ± 0.90 mmol/L, *p* < 0.05; and Ctrl T90: 0.82 ± 0.17 mmol/L *vs.* LPS T90: 3.04 ± 0.90 mmol/L, *p* < 0.001, Figure 4(b)). These results suggest a compensated state 50 min after LPS administration, followed by the onset of hypotension, which is probably a cause of the hypoperfusion and tissue distress detected during the decompensation phase at 90 min after LPS administration.

### 3.2. Identification of Deregulated Proteins in the Secretome during Sepsis

Over the 48 samples included in the study, 401 and 514 proteins were quantified by LC-MS/MS analyses in the T50 (CT and LPS) and T90 (CT and LPS) groups, respectively (Figure 2). Cleaning raw data by protein coverage > 15% and number of missing data (NA) per group led to the identification of 140 proteins at T50 and 132 proteins at T90 (Figure 2). DEP in the control groups (14) were excluded from this study. Three levels of analyses were then performed (1) the study of proteins identified in LPS samples, both in the T50 and T90 groups; (2) the study of proteins identified only in the T50 group; and (3) the study of proteins identified only in the T90 group (Figure 2).

#### 3.2.1. Study of the Evolution of the Level of Proteins Identified in the T50 and T90 Groups

Seventy-nine proteins were found to be common in the T50 and T90 groups (CT and LPS) (Figure 2 and Supplementary Table 2). The study of levels of these proteins over time showed a variation in their expression profile between the CT and LPS groups (Supplementary Figure S1-2).

Among these 79 proteins, to analyze common or deregulated proteins, T50 or T90 was compared to their T0. Among the LPS groups, 15 proteins were upregulated (5 proteins at T50 groups and 10 proteins at T90). Finally, 13 proteins were found downregulated: 1 at T50 and 12 at T90 (Table 1).

**Figure 5:**
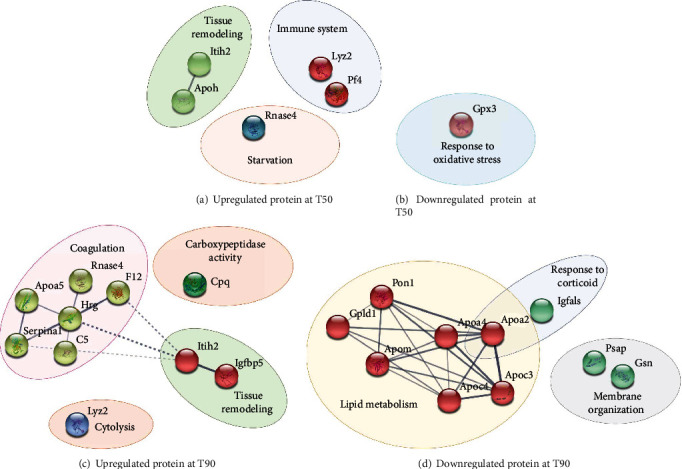
Protein-protein interaction network of (a) up and (b) downregulated proteins in the LPS T50 group and (c) up and (d) downregulated proteins in the LPS T90 groups. The study of proteins identified both in T50 (CT and LPS) and T90 (CT and LPS). The analyses using Gene Ontology (GO) databases revealed the most modified biological processes following the injection of LPS. STRING protein database was used to clustered deregulated proteins. The continuous lines represent protein interactions within the cluster, and the discontinuous lines represent protein interactions between different clusters.

The analyses of Gene Ontology highlighted functional annotations of the selected proteins. The analyses of the GO cellular component (CC) showed that the proteins identified are plasma proteins (Supplementary Table 1). Fifty minutes after LPS administration, upregulated proteins belong to tissue remodeling, immune system, and starvation (Figure 5(a)), whereas downregulated ones belong to response to oxidative stress (Figure 5(b)). Ninety minutes after LPS administration, upregulated proteins belong to carboxypeptidase activity, cytolysis, tissue remodeling, and coagulation (Figure 5(c)), whereas downregulated ones belong to lipid metabolism, membrane organization, and response to corticoid (Figure 5(d)).

#### 3.2.2. Identification of Proteins Specific to the T50 Groups

**Figure 6:**
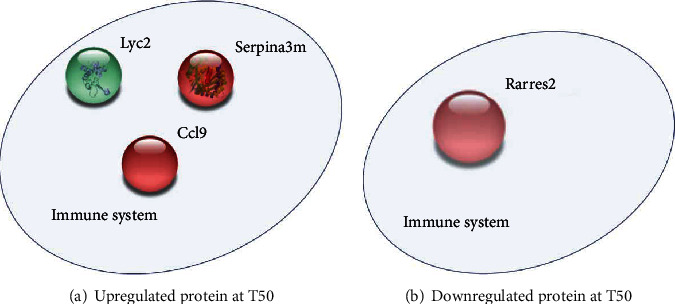
The network of (a) upregulated proteins and (b) downregulated protein in the LPS T50-specific group. The study of proteins identified in T50 (CT and LPS) and not in T90 (CT and LPS). The analyses using Gene Ontology (GO) databases reveal the most modified biological processes (BP) following the injection of LPS. STRING protein database was used to clustered deregulated proteins.

Analyses of the proteins specific to T50 (CT and LPS) identified 57 proteins that are present at T50 but not at T90 (Figure 2 and Supplementary Table 3). The study of protein expression over time revealed a variation in the protein profile between the control and LPS groups (Supplementary Figure 3). Among the 57 proteins identified, 3 are upregulated and 1 protein is downregulated 50 minutes after LPS administration (Table 2). Either upregulated or downregulated proteins specific to the T50 group are implicated in immune response (Figure 6).

#### 3.2.3. Identification of Proteins Specific to the T90 Groups

Analyses of the proteins specific to T90 (CT and LPS) identified 53 proteins that are present at T90 but not at T50 (Figure 2 and Supplementary S4). The study of protein expression over time revealed a variation in the protein expression profile between the control and LPS groups (Figure S4). Among the 53 proteins identified, 11 proteins were found to be specifically deregulated in the LPS group between T0 and T90. Among the deregulated proteins in the LPS group, 1 protein is upregulated and 10 are downregulated (Table 3).

**Figure 7:**
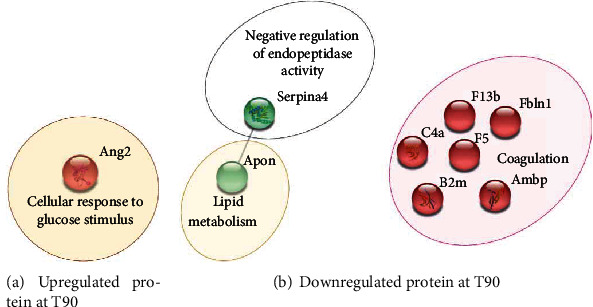
The network of (a) upregulated and (b) downregulated proteins in the LPS T90-specific group. The analyses using Gene Ontology (GO) databases revealed the most modified biological processes (BP) following the injection of LPS. STRING protein database was used to clustered deregulated proteins. The continuous line represents protein interactions within the cluster.

Upregulated proteins specific to the T90 group are implicated in glucose metabolism, whereas downregulated ones are implicated in lipid metabolism, negative regulation of endopeptidase activity, and coagulation (Figure 7).

### 3.3. Validation of Gpx3 Downregulation by Western Blotting

**Figure 8:**
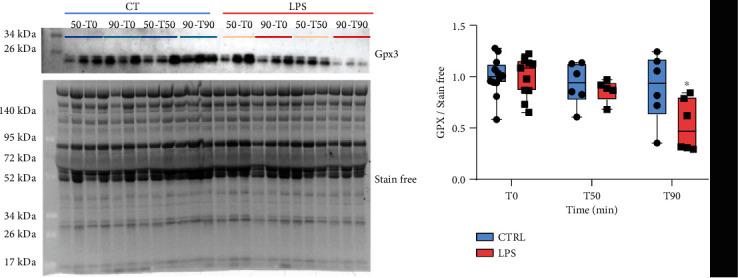
Measure in plasma level expression of glutathione peroxidase 3 (Gpx3) throughout the development of the shock. Quantification of Gpx3 levels was normalized to stain-free. Results are expressed as a mean ± SEM. ^∗^*p* < 0.05: two-way ANOVA with repeated measures and a Bonferroni post hoc test.

The antioxidant enzyme Gpx3 plays a critical role in the protection of tissues and organs from oxidative damage. Plasmatic expression levels of Gpx3 were assessed by Western blotting. No modification in plasmatic levels of Gpx3 were observed between groups at T0 and T50. Ninety minutes after shock induction, Gpx3 expression levels in the plasma were decreased by 2-fold in the LPS group compared to T0 (LPST90-T0: 1.00 ± 0.16; LPST90-T90: 0.53 ± 0.19) (Figure 8).

## 4. Discussion

Our study is aimed at identifying the secretome adaptations of a rat model of septic shock. For this purpose, a proteomic analysis was performed in the secretome of control rats and rats in endotoxemic shock 50 (T50) or 90 (T90) minutes after LPS administration. Thus, this study integrates the notion of pathology progression at 2 time points. The T50 point represents a compensated system, whereas the T90 point represents an acute phase of decompensation. Three levels of analyses have been carried out: first the study of proteins identified in both T50 and T90 groups (CT and LPS), next the study of proteins only identified in the T50 group (CT and LPS), and finally the study of proteins only identified in the T90 group (CT and LPS). All these studies demonstrate the deregulation of plasma levels of 11 proteins at 50 min and 33 proteins at 90 min after LPS administration.

### 4.1. Model of Endotoxemic Shock

In this study, we compared, at 2 different times, the evolution of protein expression during the progression of septic shock. For this, a rat model of endotoxemic shock, previously described, was chosen [32]. The endotoxemic shock model was preferred because the use of LPS allows a better control of the kinetics of the development of pathology. In this way, the sampling should lead to more homogeneous interindividual results. In this study, 90 min after the injection of LPS, the animals developed a clinical picture similar to sepsis with systemic arterial hypotension leading to an increase in plasma creatinine and lactate, which characterize organ dysfunction and altered metabolism [33]. These results show that this model of early endotoxemic shock reproduces several characteristics of septic shock. However, interestingly, the heart rate did not change after the injection of LPS. This could be explained by the strain of LPS used. Nevertheless, according to the definition of Singer and collaborators, animals can be considered in septic shock [3, 34]. The secretome of rats developing such a shock was studied by proteomic analysis based on nontargeted mass spectrometry. Analyses of the GOs of the plasma secretome showed that the state of shock leads to an alteration of several biological processes.

### 4.2. Proteomic Analyses of the Secretome

#### 4.2.1. Altered Protein Expression at T50

Fifty minutes after shock induction, DEP were identified while the physiological parameters show any signs of tissue suffering or hypoperfusion. At this time, affected biological processes are associated with the immune system, starvation, and response to oxidative stress. Our study, using a model of shock triggered by LPS injection, demonstrated an increase in beta-2-glycoprotein I (B2GPI), also known as apolipoprotein H, which is a potential regulator of complement. A recent study proposed that B2GPI may be able to mediate the anti-inflammatory effects of LPS by shunting it away from TLR4 activation [35]. This neutralization of LPS could be an effective means to limit the toxic consequences of severe Gram-negative infections. A study performed on patients admitted to the hospital following sepsis showed a decrease in B2GPI within 48 hours of patient admission [13]. A study performed in a mouse model 6 and 24 hours after induction of endotoxemic shock highlighted that the decrease in total B2GPI levels is thought to be due to the functional utilization of B2GPI as part of the protective response of the immune system [36]. Overall, these data suggest that LPS may trigger an increase in plasma B2GPI levels during the early phase of endotoxemic shock that would later on be consumed which would explain the decrease of B2GPI described in the literature. These results suggest that decreased B2GPI could be a biomarker of the early phase of sepsis.

#### 4.2.2. Altered Protein Expression at T90

Ninety minutes after shock induction, this study identified deregulated proteins involved in biological processes associated with coagulation and lipid metabolism. This study demonstrated the deregulation of apolipoprotein (Apo) expression which is related to the metabolism of high-density lipoproteins (HDL). In this study, analyses of the secretome in the LPS group at 90 minutes show a decrease in Apo N, Apo M, Apo C-III, Apo C-IV, Apo A-IV, and Apo A-II and an increase in Apo AV. Among these proteins, some are not yet described, such as Apo C-III and Apo C-IV, while others have already been identified in sepsis. These data reinforce the interest of our study. Khovidhunkit and colleagues described a decrease in hepatic mRNA levels of Apo A-II and an increase in Apo A-V mRNA 8 hours after endotoxin injection. These changes in hepatic mRNA levels lead to a decrease of Apo A-II and an increase of Apo A-V in HDL particles [20]. Interestingly, a recent study suggests, in septic pediatric patients, the prognostic value of Apo A-V based on an observation of an increase in Apo A-V levels in the serum at the admission and a decrease in patients who did not survive. Moreover, they highlighted a significant association between low levels of Apo A-V and sepsis-induced acute kidney injuries [18]. These results and those provided in this study suggest that Apo A-V could be a biomarker of septic shock. On the other hand, although studies have shown that Apo A-II is an important predictor of cardiovascular disease risk, its role in lipid metabolism is less clear and requires further research [37]. Interestingly, Apo A-II and Apo M have been described as being decreased in patients developing severe forms of COVID-19 which may be at the origin of septic shock [38].

#### 4.2.3. Altered Protein Expression at T50 and T90

Interestingly, among the deregulated proteins identified in both T50 and T90 groups, the majority of DEP identified at T50 were found to be downregulated at T90. Our study shows a decrease in the plasma expression of glutathione peroxidase 3 (Gpx3). Some studies support the major role of reactive oxygen species (ROS) in the pathogenesis of sepsis and its contribution to the progression to multiple organ dysfunction [39]. Indeed, oxidative stress caused by inflammatory response can lead to lipid peroxidation, DNA damage, and altered mitochondrial function promoting organ dysfunction. The metabolism of glutathione is an essential mechanism of antioxidant defense. The main function of Gpx3 is to catalyze the reduction of hydrogen peroxide, organic peroxides, and lipid peroxides by converting reduced glutathione into oxidized glutathione. Considering the acute oxidative stress observed in sepsis patients, it is interesting to observe that Gpx3 bioactivity is inversely associated with the severity of sepsis and associated mortality [40, 15]. Thus, although understanding of the mechanisms of regulation of Gpx3 expression and its pathophysiological role in sepsis is limited, Gpx3 could be a promising biomarker for assessing oxidative stress.

### 4.3. Limitations of the Study

The use of an endotoxemic rat model to mimic septic shock raises concern regarding the extrapolation to human. A rat model based on LPS injection does not reflect exactly the complexity of human pathophysiological responses particularly because it is based on the use of a single bacterial strain. Septic shock can be induced by several bacterial strains or even by nonbacterial pathogens. The changes in the secretome observed in this study may in part differ with the infectious agent causing the pathology, which needs to be further investigated [41]. However, it is currently the only model to follow sepsis kinetic over time in a reproducible manner. A second limitation of this study is based on the study of the plasma secretome, which requires the use of selective depletion techniques. The plasma secretome is made up of a vast dynamic range of compound concentrations. This makes analysis of proteins of low abundance particularly difficult and hinders the identification of biomarkers using mass spectrometry. A selective depletion technique, called ProteoMiner®, was used in this study to detect the low abundance protein signal in a complex protein sample by reducing the dynamic concentration range of the proteins. Although necessary, this approach may remove low abundance or low molecular weight proteins because of a weak or rare interaction between the sample proteins and the hexapeptides in this ProteoMiner®. A third limitation of this study is the validation of the DEP obtained from the proteomic analysis. Indeed, the Western blot analysis was complicated by the lack of sensitivity of this approach and by the fact that most of the antibodies tested lack specificity with the proteins identified in this study.

## 5. Conclusion

This preliminary study found several deregulated proteins at 50 and 90 minutes after LPS injection. Gene Ontology study showed that the expression of proteins associated with the response to oxidative stress, the immune system, the coagulation, or the lipid metabolism is particularly deregulated during the development of sepsis. Other studies have also reported the deregulation of these different biological processes, which reinforces the results of our work. However, this study identified proteins, such as Apo A-II or Apo C-IV, which were not described previously in sepsis phenotype. Subsequently, the proteins identified as being deregulated in this study will have to be (i) validated by the use of more complex models for a better recapitulation of what happens at the clinical level and (ii) tested on cellular models in order to understand the role they could play in the pathophysiology of shock.

## Figures and Tables

**Table 1:** Proteins with a Log_2_foldchange (Log_2_FC) greater than 1 are highlighted in bold, and proteins with a Log_2_FC less than -1 are not in bold.

UniProt accession number	Protein	Gene name	Protein reference described	Log_2_FC Median (min; max)	*n*/6
LPS T50					
**P06765**	**Platelet factor 4** ^∗^	***Pf4***	**[** 12 **]**	**3.31 (1.02; 5.20)**	**5/6**
**D3ZFH5**	**Inter-alpha-trypsin inhibitor heavy chain 2**	***Itih2***	**—**	**1.25 (1.08; 2.34)**	**4/6**
**P26644**	**Beta-2-glycoprotein 1** ^∗^	***Apoh*** ***B2GPI***	**[** 13 **]**	**1.45 (1.03; 1.55)**	
**O55004**	**Ribonuclease 4**	***RNase 4***	**—**	**1.78 (1.70; 2.90)**	
**P00697**	**Lysozyme C-1** ^∗^	***Lyz1***	**[** 14 **]**	**1.77 (1.70; 2.63)**	
P23764	Glutathione peroxidase 3^∗^	*Gpx3*	[15]	-2.58 (-1.45; -4.75)	4/6

LPS T90					
**P17475**	**Alpha-1-antiproteinase** ^∗^	***Serpina1***	**[** 16 **]**	**3.73 (2.97; 5.65)**	**5/6**
**Q99PS8**	**Histidine-rich glycoprotein** ^∗^	***Hrg***	**[** 17 **]**	**2.39 (1.37; 3.89)**	
**D3ZFH5**	**Inter-alpha-trypsin inhibitor heavy chain 2**	***Itih2***	**—**	**1.92 (1.58; 3.82)**	**4/6**
**Q9QUH3**	**Apolipoprotein A-V** ^∗^	***Apoa5***	**[** 18 **]**	**1.44 (1.00; 2.24)**	
**P24594**	**Insulin-like growth factor-binding protein 5**	***Igfbp5***	**—**	**2.14 (1.02; 3.79)**	
**P08650**	**Complement C5** ^∗^	***C5***	**—**	**5.27 (3.28; 6.21)**	
**O55004**	**Ribonuclease 4**	***Rnase4***	**—**	**1.68 (1.28; 2.32)**	
**D3ZTE0**	**Coagulation factor XII** ^∗^	***F12***	**[** 19 **]**	**1.42 (1.32; 1.73)**	
**P00697**	**Lysozyme C-1** ^∗^	***Lyz1***	**[** 14 **]**	**2.86 (1.25; 3.81)**	
**Q6IRK9**	**Carboxypeptidase Q**	***Cpq***	**—**	**1.94 (1.05; 3.34)**	
P04638	Apolipoprotein A-II^∗^	*Apoa2*	[20]	-1.96 (-1.19; -5.20)	5/6
P55797	Apolipoprotein C-IV	*Apoc4*	—	-1.88 (-1.37; -2.91)	
P20767	Ig lambda-2 chain C region	*N/A*	—	2.39 (-1.50; -3.30)	
Q8R2H5	Phosphatidylinositol-glycan-specific phospholipase D^∗^	*Gpld1*	[21]	-1.48 (-1.02; -2.72)	
P14630	Apolipoprotein M^∗^	*Apom*	[22]	-2.94 (-1.46; -4.82)	
P23764	Glutathione peroxidase 3^∗^	*Gpx3*	[15]	-2.05 (-1.48; -3.44)	
Q68FP1	Gelsolin^∗^	*Gsn*	[23]	-2.05 (-1.67; -4.47)	4/6
P06759	Apolipoprotein C-III	*Apoc3*	—	-2.60 (-1.08; -3.66)	
P55159	Serum paraoxonase/arylesterase 1^∗^	*Pon1*	[24]	-2.15 (-1.17; -2.49)	
P02651	Apolipoprotein A-IV	*Apoa4*	—	-1.54 (-1.04; -2.61)	
P35859	Insulin-like growth factor-binding protein complex acid labile subunit	*Igfals*	—	-2.49 (-2.20; -2.90)	
P10960	Prosaposin^∗^	*Psap*	[25]	-2.57 (-1.53; -3.90)	

*N* represent the number of rats in which the protein has been found to be deregulated over the 6 rats. Proteins with an asterisk have already been described by other reports in the context of sepsis and septic shock.

**Table 2:** Proteins with a Log_2_foldchange (Log_2_FC) greater than 1 are highlighted in bold, and proteins with a Log_2_FC less than -1 are not in bold.

UniProt accession number	Protein	Gene name	Protein reference described	Log_2_FC (range)	*n*/6
**Q5FVN3**	**Ccl9-like protein** ^∗^	***Ccl9***	[26]	**3.69 (1.38; 4.98)**	**4/6**
**Q05820**	**Putative lysozyme C-2**	***Lyz2***	**—**	**1.67 (1.30; 2.64)**	
**Q63556**	**Serine protease inhibitor A3M**	***Serpina3m***	**—**	**1.10 (1.30; 2.63)**	
Q5BK77	Chemerin^∗^	*Rarres2*	[27]	-2.24 (-1.36; -2.98)	5/6

*N* represent the number of rats in which the protein has been found to be deregulated over the 6 rats. Proteins with an asterisk have already been described in the context of sepsis and septic shock.

**Table 3:** Proteins with a Log_2_foldchange (Log_2_FC) greater than 1 are highlighted in bold, and proteins with a Log_2_FC less than -1 are not in bold.

UniProt accession number	Protein	Gene name	Protein reference described	Log_2_FC (range)	*n*/6
**Q5GAM5**	**Angiogenin ribonuclease 2**	***Ang2***	**—**	**1.79 (1.32; 3.47)**	**4/6**
Q6MG90	C4a anaphylatoxin	*C4a*		-3.22 (-1.42; -6.85)	6/6
D3ZQ25	Fibulin-1	*Fbln1*		-2.37 (1.24; 5.70)	
Q5M890	Apolipoprotein N	*Apon*	—	-4.55 (-1.46; -5.61)	
Q5M8C3	Serine (or cysteine) proteinase inhibitor, clade A (alpha-1 antiproteinase, antitrypsin), member 4	*Serpina4*	—	-1.59 (-1.11; -2.75)	
Q64240	Protein AMBP^∗^	*Ambp*	[28]	-3.25 (-1.09; -5.11)	
B1H260	Coagulation factor XIII B chain	*F13b*	—	-3.86 (-1.07; -5.12)	5/6
F1LZ11	Ig-like domain-containing protein	*N/A*	—	-1.72 (-1.00; -3.56)	
A0A0G2JXI1	Coagulation factor V^∗^	*F5*	[29]	-2.23 (-1.76; -4.25)	4/6
P08649	Complement C4^∗^	*C4*	[30]	-3.14 (-1.16; -4.59)	
P07151	Beta-2-microglobulin^∗^	*B2m*	[31]	-1.51 (-1.32; -2.36)	

*N* represent the number of rats in which the protein has been found to be deregulated over the 6 rats. Proteins with an asterisk have already been described in the context of sepsis and septic shock.

## Data Availability

The proteomic data were deposited to the ProteomeXchange Consortium with the MassIVE identifier MSV000081840 (http://massive.ucsd.edu) and ProteomeXchange identifier PXD008530 (http://www.proteomexchange.org).

## Supplementary Materials

Supplementary Materials Supplementary Figure 1 represents the variation in fold change of each protein, common to the T90 and T50 groups, identified in the control and LPS group 50 minutes after shock induction. Supplementary Figure 2 represents the variation of the fold change of each protein, common to the T90 and T50 groups, identified in the control and LPS group 90 minutes after shock induction. Supplementary Figure 3 represents the variation in fold change of each protein, specific to the T50 group, identified in the control and LPS group 50 minutes after shock induction. Supplementary Figure 4 represents the variation in fold change of each protein, specific to the T90 group, identified in the control and LPS group 90 minutes after shock induction. Supplementary Table 1 corresponds to the results of Gene Ontology (GO) analysis which highlighted the most modified cellular components (CC) following the injection of LPS. Supplementary Table 2 includes all quantified proteins and unique peptides as well as measured coverage and calculated Log_2_foldchange for proteins common to the T50 and T90 groups. Supplementary Table 3 includes all quantified proteins, unique peptides, and the measured coverage and calculated Log_2_foldchange for T50-specific proteins. Supplementary Table 4 includes all quantified proteins, unique peptides, and the measured coverage and calculated Log2foldchange for T90-specific proteins.
