# Supplementary material for: The Secretome Deregulations in a Rat Model of Endotoxemic Shock
Source: Oxid Med Cell Longev. 2021 Jul 24;2021:6650464. doi: 10.1155/2021/6650464 (PMC8328724; doi:10.1155/2021/6650464)
Supplement: Supplementary Materials — Supplementary Figure 1 represents the variation in fold change of each protein, common to the T90 and T50 groups, identified in the control and LPS group 50 minutes after shock induction. Supplementary Figure 2 represents the variation of the fold change of each protein, common to the T90 and T50 groups, identified in the control and LPS group 90 minutes after shock induction. Supplementary Figure 3 represents the variation in fold change of each protein, specific to the T50 group, identified in the control and LPS group 50 minutes after shock induction. Supplementary Figure 4 represents the variation in fold change of each protein, specific to the T90 group, identified in the control and LPS group 90 minutes after shock induction. Supplementary Table 1 corresponds to the results of Gene Ontology (GO) analysis which highlighted the most modified cellular components (CC) following the injection of LPS. Supplementary Table 2 includes all quantified proteins and unique peptides as well as measured coverage and calculated Log2foldchange for proteins common to the T50 and T90 groups. Supplementary Table 3 includes all quantified proteins, unique peptides, and the measured coverage and calculated Log2foldchange for T50-specific proteins. Supplementary Table 4 includes all quantified proteins, unique peptides, and the measured coverage and calculated Log2foldchange for T90-specific proteins. [file 6650464.f1.zip › Supplemental Figures and table 1 (1).pdf]

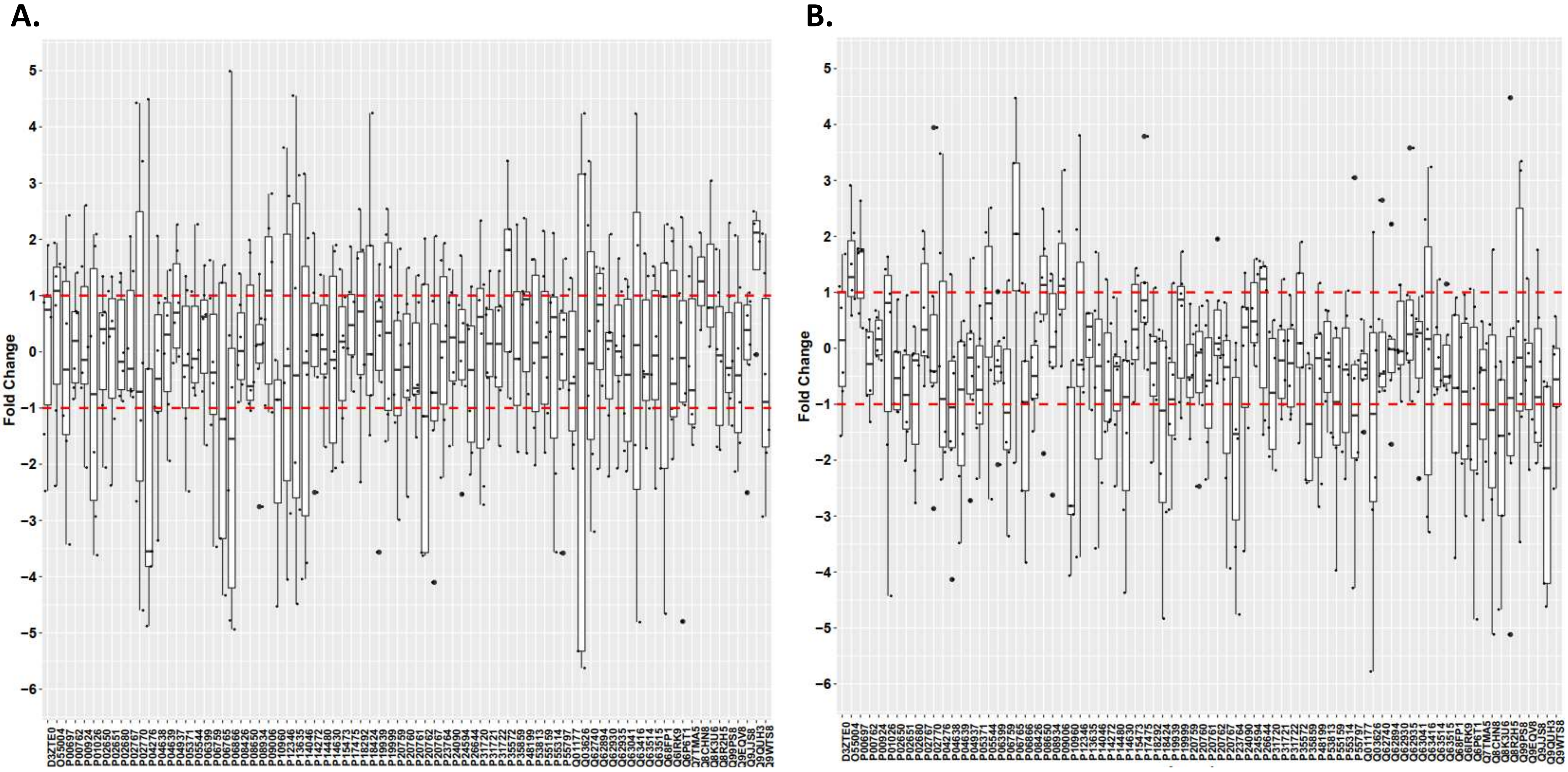

Supplemental Figure 1: Barplot of plasmatic levels of proteins identified **A.** Control T50 **B.** LPS T50

The analysis of the variation in plasmatic protein levels between T0 and T50 was performed with the Log_2 of the T50/T0 ratio (FC) for each protein in the LPS and control groups. Each point represents the plasma level of the protein for each rat in the group. The red dashed lines represent the thresholds for which proteins are up ($Log_2FC > 1$) or downregulated ($Log_2FC < -1$).

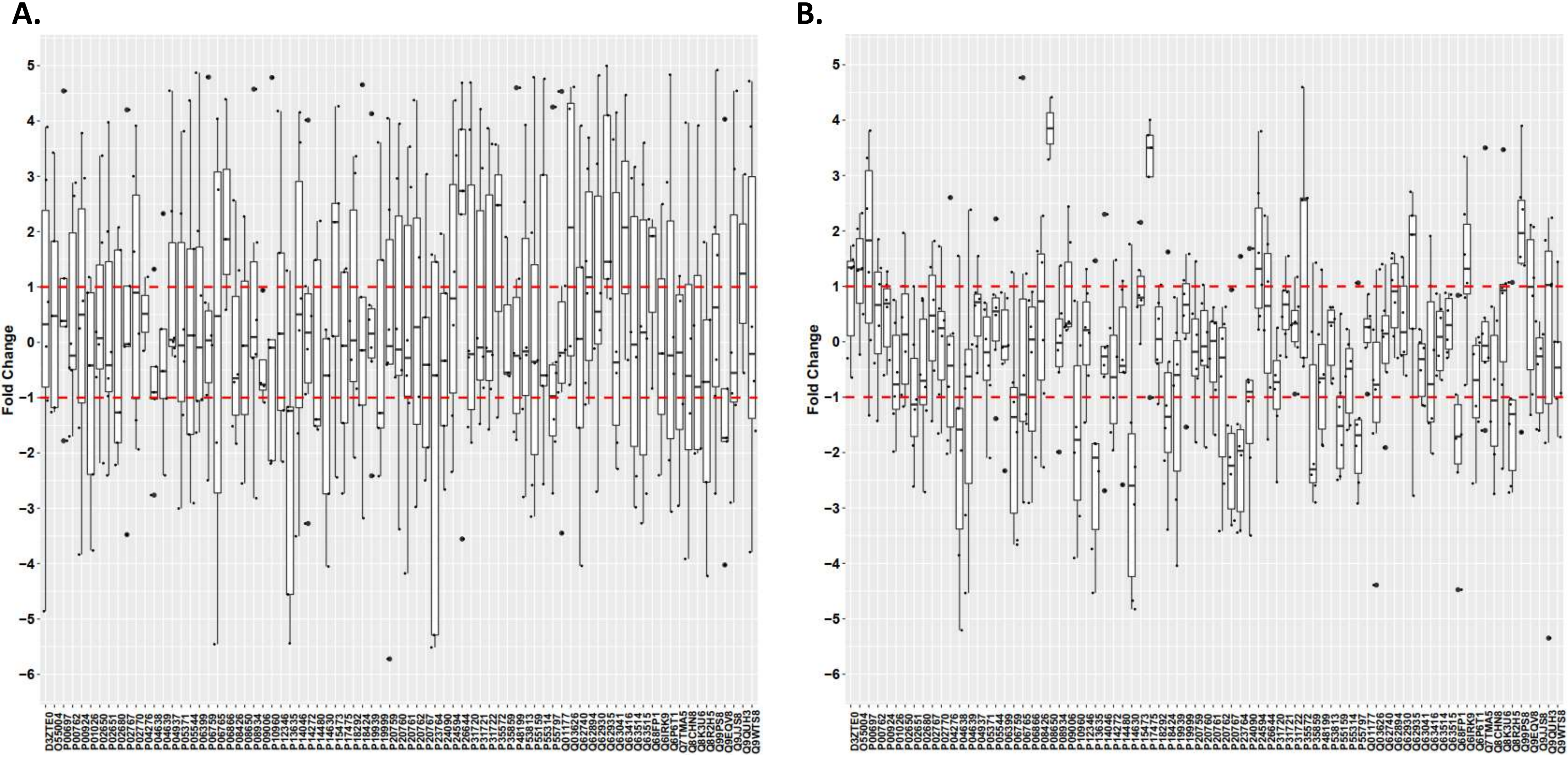

Supplemental Figure 2: Barplot of proteins identified **A.** Control T90 **B.** LPS T90

The analysis of the variation in plasmatic protein levels between T0 and T50 was performed with the Log_2 of the T90/T0 ratio (FC) for each protein in the LPS and control groups. Each point represents the plasma level of the protein for each rat in the group. The red dashed lines represent the thresholds for which proteins are up ($Log_2FC > 1$) or downregulated ($Log_2FC < -1$).

LPS T50							
#term ID	term description	observed gene count	background gene count	strength	false discovery rate	matching proteins in your network (IDs)	matching proteins in your network (labels)
GO:0005576	extracellular region	3	1052	1.11	0.0352	10116.ENSRNOP00000007747,10116.ENSRNOP00000032276,10116.ENSRNOP00000043003	Lyz2,Pf4,Rnase4
GO:0030141	secretory granule	2	217	1.62	0.0352	10116.ENSRNOP00000007747,10116.ENSRNOP00000032276	Lyz2,Pf4
LPS T90							
GO:0005576	extracellular region	9	1052	1.29	5.09e-10	10116.ENSRNOP00000007747,10116.ENSRNOP00000008211,10116.ENSRNOP00000012577,10116.ENSRNOP00000023530,10116.ENSRNOP00000024918,10116.ENSRNOP00000025534,10116.ENSRNOP00000043003,10116.ENSRNOP00000049290,10116.ENSRNOP00000061983	Lyz2,Cpq,Serpina1,Igfbp5,Apoa5,C5,Rnase4,Hrg,F12
GO:0005615	extracellular space	8	631	1.46	5.09e-10	10116.ENSRNOP00000007747,10116.ENSRNOP00000008211,10116.ENSRNOP00000012577,10116.ENSRNOP00000023530,10116.ENSRNOP00000024918,10116.ENSRNOP00000025534,10116.ENSRNOP00000049290,10116.ENSRNOP00000061983	Lyz2,Cpq,Serpina1,Igfbp5,Apoa5,C5,Hrg,F12
GO:0072562	blood microparticle	2	17	2.43	0.00052	10116.ENSRNOP00000024918,10116.ENSRNOP00000049290	Apoa5,Hrg
GO:0034364	high-density lipoprotein particle	6	14	2.99	2.64e-15	10116.ENSRNOP00000001126,10116.ENSRNOP00000004662,10116.ENSRNOP00000011823,10116.ENSRNOP00000024196,10116.ENSRNOP00000024832,10116.ENSRNOP00000067045	Apom,Apoa2,Pon1,Gpld1,Apoc4,Apoc3
GO:0005615	extracellular space	9	631	1.51	1.27e-12	10116.ENSRNOP00000000696,10116.ENSRNOP00000001126,10116.ENSRNOP00000004662,10116.ENSRNOP00000011823,10116.ENSRNOP00000020233,10116.ENSRNOP00000024196,10116.ENSRNOP00000024832,10116.ENSRNOP00000025857,10116.ENSRNOP00000067045	Psap,Apom,Apoa2,Pon1,Igfals,Gpld1,Apoc4,Gsn,Apoc3
GO:0034366	spherical high-density lipoprotein particle	4	6	3.18	4.13e-11	10116.ENSRNOP00000001126,10116.ENSRNOP00000004662,10116.ENSRNOP00000011823,10116.ENSRNOP00000067045	Apom,Apoa2,Pon1,Apoc3
GO:0034361	very-low-density lipoprotein particle	4	13	2.85	3.50e-10	10116.ENSRNOP00000001126,10116.ENSRNOP00000004662,10116.ENSRNOP00000024832,10116.ENSRNOP00000067045	Apom,Apoa2,Apoc4,Apoc3
GO:0032991	protein-containing complex	8	2124	0.93	1.39e-06	10116.ENSRNOP00000001126,10116.ENSRNOP00000004662,10116.ENSRNOP00000011823,10116.ENSRNOP00000020233,10116.ENSRNOP00000024196,10116.ENSRNOP00000024832,10116.ENSRNOP00000025857,10116.ENSRNOP00000067045	Apom,Apoa2,Pon1,Igfals,Gpld1,Apoc4,Gsn,Apoc3
GO:0042627	chylomicron	2	8	2.76	4.46e-05	10116.ENSRNOP00000004662,10116.ENSRNOP00000067045	Apoa2,Apoc3

Table Supplemental 1: Analyses of cellular GO cellular component of common deregulated proteins at 50 and 90 minutes after induction of endotoxemic shock. Analysis using Gene Ontology (GO) databases has made it possible to identify the cellular components (CC) most modified as a result of the injection of LPS.

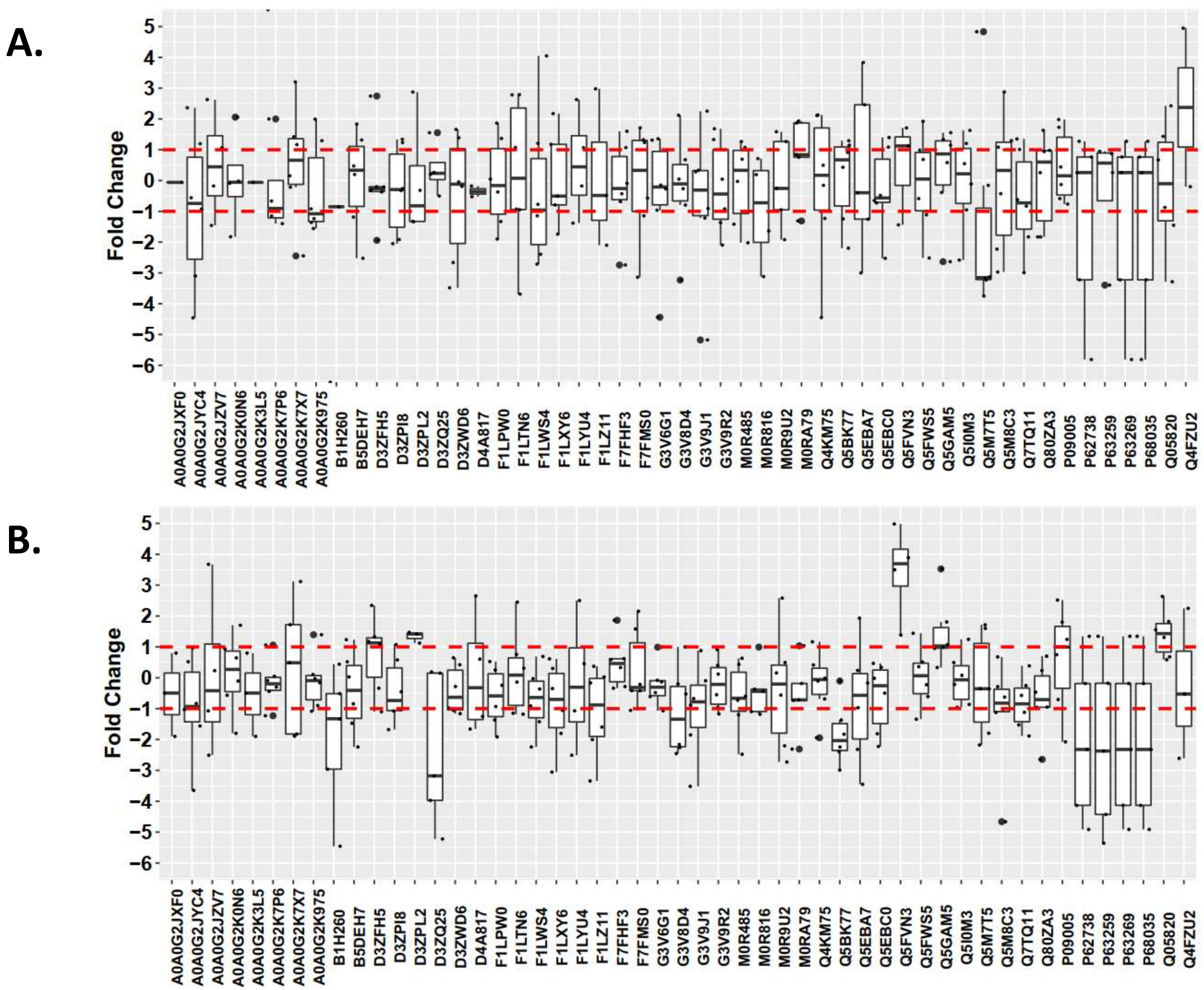

Supplemental Figure 3: Barplot of proteins specific to T50 group identified **A.** Control T50 **B.** LPS T50

The analysis of the variation in plasmatic protein levels between T0 and T50 was performed with the Log_2 of the T50/T0 ratio (FC) for each protein in the LPS and control groups. Each point represents the plasma level of the protein for each rat in the group. The red dashed lines represent the thresholds for which proteins are up ($Log_2FC > 1$) or downregulated ($Log_2FC < -1$).

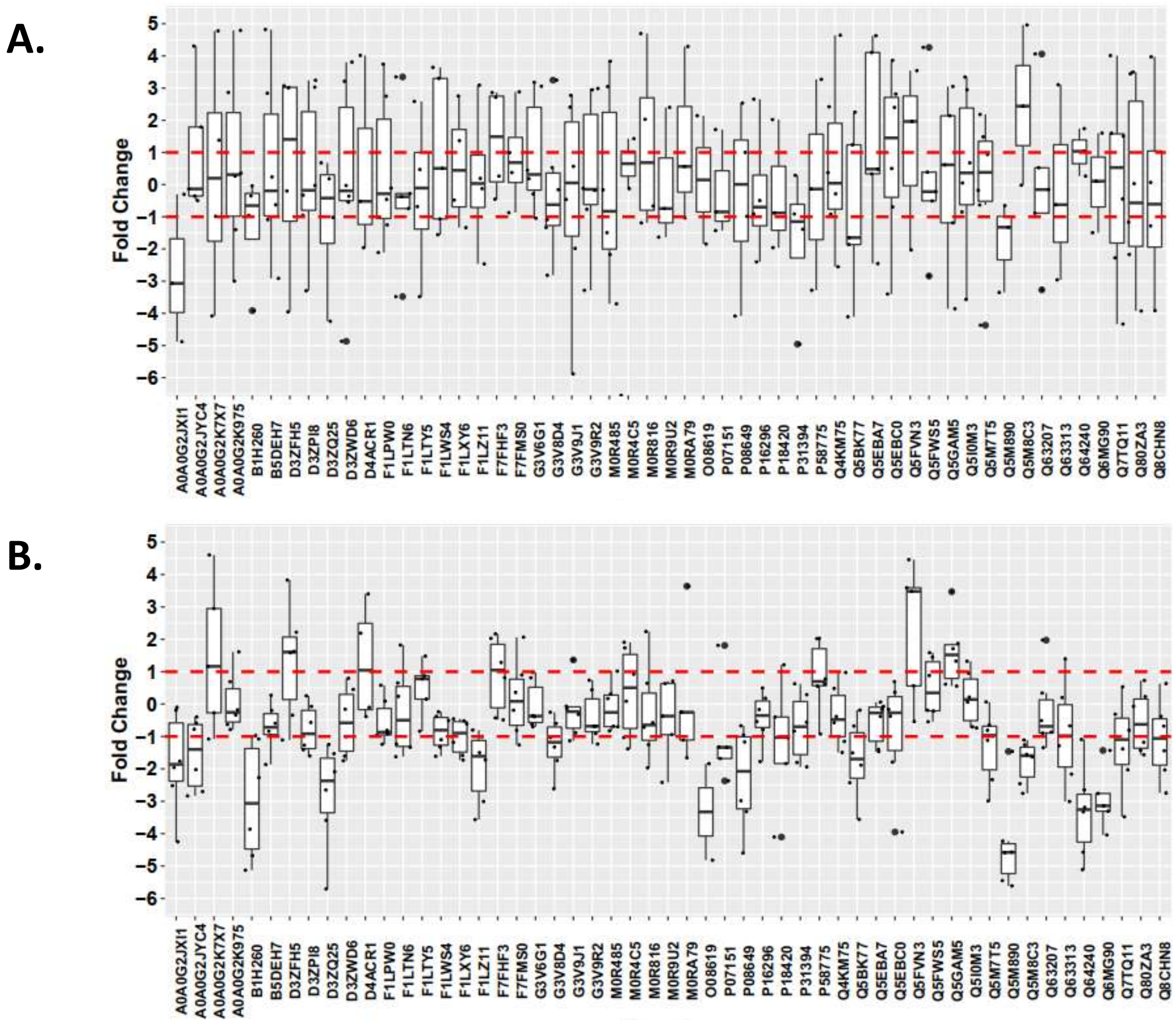

Supplemental Figure 4: Barplot of proteins specific to T90 group identified **A.** Control T90 **B.** LPS T90.

The analysis of the variation in plasmatic protein levels between T0 and T50 was performed with the Log_2 of the T90/T0 ratio (FC) for each protein in the LPS and control groups. Each point represents the plasma level of the protein for each rat in the group. The red dashed lines represent the thresholds for which proteins are up ($Log_2FC > 1$) or downregulated ($Log_2FC < -1$).
