# Supplementary material for: The Secretome Deregulations in a Rat Model of Endotoxemic Shock
Source: Oxid Med Cell Longev. 2021 Jul 24;2021:6650464. doi: 10.1155/2021/6650464 (PMC8328724; doi:10.1155/2021/6650464)
Supplement: Supplementary Materials — Supplementary Figure 1 represents the variation in fold change of each protein, common to the T90 and T50 groups, identified in the control and LPS group 50 minutes after shock induction. Supplementary Figure 2 represents the variation of the fold change of each protein, common to the T90 and T50 groups, identified in the control and LPS group 90 minutes after shock induction. Supplementary Figure 3 represents the variation in fold change of each protein, specific to the T50 group, identified in the control and LPS group 50 minutes after shock induction. Supplementary Figure 4 represents the variation in fold change of each protein, specific to the T90 group, identified in the control and LPS group 90 minutes after shock induction. Supplementary Table 1 corresponds to the results of Gene Ontology (GO) analysis which highlighted the most modified cellular components (CC) following the injection of LPS. Supplementary Table 2 includes all quantified proteins and unique peptides as well as measured coverage and calculated Log2foldchange for proteins common to the T50 and T90 groups. Supplementary Table 3 includes all quantified proteins, unique peptides, and the measured coverage and calculated Log2foldchange for T50-specific proteins. Supplementary Table 4 includes all quantified proteins, unique peptides, and the measured coverage and calculated Log2foldchange for T90-specific proteins. [file 6650464.f1.zip › Supplemental table 3- proteins specific T50 groups (1).pdf]

Accession	Unique peptide	Coverage	Area.C50.T0.1	Area.C50.T0.3	Area.C50.T0.4	Area.C50.T0.5	Area.C50.T0.6	Area.C50.T0.7	Area.C50.T50.1	Area.C50.T50.3	Area.C50.T50.4	Area.C50.T50.5	Area.C50.T50.6	Area.C50.T50.7
P20059	34	63	502000	56400000	7,00E+05	236000	18900000	194000	22900000	84000	64300	1,50E+07	860000	342000
Q9QX79	21	56	241000	6640000	NA	NA	2660000	123000	2680000	143000	17700	2890000	64400	26100
M0RA79	4	42	61800	536000	126000	72200	3080000	38400	236000	216000	460000	122000	5510000	NA
M0R816	3	22	78100	934000	112000	NA	224000	NA	128000	3,00E+05	128000	156000	25800	NA
A0A0G2K0N6	6	63	117000	872000	NA	77000	135000	39200	110000	246000	11800	321000	133000	NA
F7FHF3	11	16	2300000	3620000	706000	2770000	3750000	1920000	2160000	2690000	2140000	5810000	2330000	287000
P09005	11	42	6320000	5640000	1990000	6720000	4450000	341000	5770000	7650000	7850000	4480000	2620000	1130000
D4A817	5	37	689000	1380000	328000	NA	841000	NA	608000	959000	NA	177000	NA	0
D3ZFH5	26	32	3990000	5660000	742000	2370000	3430000	324000	3470000	4540000	4970000	2050000	894000	251000
G3V8D4	32	80	44800000	1,50E+08	39900000	46500000	64700000	1,66E+08	37300000	86500000	63700000	2,02E+08	66800000	17700000
D3ZPL2	8	38	502000	2190000	NA	792000	193000	NA	403000	868000	NA	5800000	77000	NA
Q5GAM5	14	64	3140000	3450000	1390000	4560000	2730000	4400000	2400000	7630000	3510000	13400000	4090000	709000
Q5EBC0	67	74	1,80E+07	30800000	6790000	8050000	12400000	10200000	12900000	18600000	14400000	21200000	7900000	1770000
P01015	13	32	1190000	1450000	325000	709000	1140000	NA	761000	1910000	1740000	757000	317000	30000
F1LTN6	34	59	1,80E+07	29900000	6220000	4090000	2210000	2410000	9470000	15500000	13100000	28300000	15200000	187000
M0R9U2	3	23	1160000	684000	242000	174000	266000	20000	6,00E+05	573000	582000	522000	70300	NA
Q5I0M3	32	65	34300000	30600000	11500000	15600000	1,90E+07	30100000	17700000	28300000	26500000	48200000	27800000	5030000
G3V6G1	9	48	5610000	7470000	1300000	3830000	3880000	2280000	2880000	6190000	3330000	9510000	3500000	105000
P08025	4	41	848000	30400	NA	NA	390000	2270000	411000	342000	120000	946000	1540000	57600
Q5FWS5	32	59	10500000	8930000	2490000	4750000	6370000	5590000	4850000	17700000	9450000	7630000	4240000	978000
F7FMS0	17	27	1060000	619000	271000	918000	892000	1120000	479000	1550000	888000	2160000	6,00E+05	127000
B5DEH7	65	75	19500000	23200000	5900000	7610000	6510000	17100000	8570000	26600000	14700000	27100000	9040000	2970000
G3V9J1	69	57	4760000	6950000	2640000	2030000	1690000	7230000	2050000	3710000	3330000	9680000	2060000	2,00E+05
Q63556	16	40	5490000	1830000	840000	3570000	1730000	150000	2350000	2690000	3990000	2180000	1570000	121000
Q4KM75	14	38	5090000	6700000	548000	2380000	2650000	2110000	2170000	6010000	2370000	10400000	3740000	96700
Q5BK77	12	55	3390000	2320000	1390000	3590000	2190000	3350000	1430000	4370000	3080000	8800000	2950000	731000
F1LPW0	49	60	1,15E+08	1,24E+08	23800000	27200000	42800000	19100000	45700000	1,28E+08	61400000	9,90E+07	33100000	5120000
G3V9R2	245	91	5,53E+08	4,77E+08	1,22E+08	1,35E+08	2,50E+08	2,59E+08	2,14E+08	2,52E+08	3,07E+08	4,33E+08	2,58E+08	60600000
F1LYU4	3	23	352000	322000	66000	81700	106000	26200	135000	285000	408000	171000	NA	NA
A0A0G2K7P6	5	64	348000	NA	0	59500	119000	45000	132000	374000	166000	238000	75100	20300
M0R485	35	59	44900000	22100000	13400000	18500000	1,10E+07	16400000	16800000	35700000	32200000	3,90E+07	10800000	4040000
Q5FVN3	14	79	797000	NA	NA	22500	173000	267000	294000	621000	116000	73600	377000	NA
Q05820	7	40	435000	1070000	147000	840000	482000	466000	159000	1700000	790000	2270000	263000	47800
A0A0G2JZV7	10	63	371000	322000	66000	81700	106000	118000	135000	285000	408000	171000	NA	NA
A0A0G2K975	79	72	19300000	19700000	5300000	8930000	16800000	19900000	6560000	8350000	1,30E+07	35500000	8870000	7750000
F1LXY6	7	84	2650000	1990000	105000	474000	335000	76000	792000	1150000	474000	1070000	236000	NA
Q7TQ11	24	32	20400000	14800000	3300000	5610000	10900000	11300000	5740000	9610000	8420000	11300000	6130000	1420000
Q80ZA3	49	74	24400000	9480000	4810000	6970000	2980000	5160000	6860000	18700000	14900000	13400000	3560000	1450000
D3ZPI8	12	80	3750000	6160000	2810000	817000	3660000	4180000	994000	4920000	7100000	1920000	3050000	1010000
F1LZ11	7	58	480000	424000	9470	NA	NA	16000	112000	303000	74600	186000	NA	NA
F1LWS4	25	44	7620000	4930000	1,00E+06	159000	1680000	2120000	1450000	2230000	2310000	2630000	983000	323000
A0A0G2K7X7	15	19	807000	274000	49200	428000	106000	89100	148000	615000	453000	476000	284000	76700
D3ZWD6	48	77	7850000	12900000	5280000	1290000	8160000	11100000	1240000	11300000	16700000	3380000	7910000	992000
Q5M8C3	22	56	13400000	2580000	585000	249000	1380000	31400	1710000	1920000	4300000	612000	295000	66500
Q5EBA7	12	22	706000	1070000	73800	81300	372000	1060000	88300	813000	406000	1160000	156000	NA
A0A0G2JYC4	19	48	1790000	1030000	120000	183000	513000	679000	209000	542000	275000	942000	348000	30900
P68035	11	23	3130000	2580000	532000	1090000	2180000	235000	335000	6240000	NA	1850000	38800	281000
P68136	11	23	3130000	2580000	532000	1090000	2180000	235000	335000	6240000	NA	1850000	38800	281000
P62738	11	23	3130000	2580000	532000	1090000	2180000	235000	335000	6240000	NA	1850000	38800	281000
P63269	11	23	3130000	2580000	532000	1090000	2180000	235000	335000	6240000	NA	1850000	38800	281000
P63259	13	36	4800000	3550000	664000	1090000	2600000	235000	456000	6860000	NA	2,00E+06	38800	281000
P60711	13	36	4800000	3550000	664000	1090000	2600000	235000	456000	6860000	NA	2,00E+06	38800	281000
Q5M7T5	26	54	33400000	7460000	613000	575000	9500000	2260000	2480000	796000	550000	16400000	1050000	259000
B1H260	23	36	65200	296000	NA	NA	152000	85900	NA	164000	46900	4550000	0	NA
D3ZQ25	20	30	178000	680000	53500	145000	48800	181000	NA	480000	157000	174000	56300	NA
Q9EQV9	10	24	348000	357000	50500	468000	409000	97000	NA	613000	153000	610000	101000	NA
Q6QMY6	4	16	45700	37600	NA	270000	NA	60500	NA	172000	181000	60000	209000	NA

Accession	Area.L50.T0.1	Area.L50.T0.10	Area.L50.T0.11	Area.L50.T0.12	Area.L50.T0.13	Area.L50.T0.9	Area.L50.T50.1	Area.L50.T50.10	Area.L50.T50.11	Area.L50.T50.12	Area.L50.T50.13	Area.L50.T50.9
P20059	363000	283000	3130000	66600	4060000	93600	4980000	NA	824000	32300000	2110000	38000
Q9QX79	221000	30600	263000	251000	265000	NA	303000	NA	55800	6730000	126000	NA
M0RA79	158000	459000	285000	250000	194000	309000	139000	92600	172000	NA	118000	634000
M0R816	153000	335000	309000	232000	338000	94700	305000	157000	NA	171000	149000	73300
A0A0G2K0N6	282000	231000	1010000	383000	146000	257000	441000	751000	939000	110000	98600	492000
F7FHF3	1590000	1500000	4930000	3910000	7780000	484000	5780000	1890000	4050000	5890000	6160000	744000
P09005	2130000	2330000	1650000	4070000	4940000	1010000	12200000	553000	2770000	14200000	3030000	2390000
D4A817	1150000	401000	NA	405000	NA	115000	364000	2520000	119000	170000	309000	175000
D3ZFH5	1200000	372000	2890000	2050000	3780000	563000	6090000	788000	1350000	5150000	2990000	1270000
G3V8D4	2,43E+08	4,79E+08	6,17E+08	3,08E+08	4,63E+08	15400000	1,69E+08	87400000	1,29E+08	68900000	4,03E+08	30800000
D3ZPL2	NA	465000	218000	182000	110000	237000	297000	NA	475000	486000	NA	656000
Q5GAM5	6790000	4740000	6460000	5810000	1,50E+07	304000	23800000	9410000	12500000	12200000	18900000	3500000
Q5EBC0	2,50E+07	46700000	70600000	30500000	57700000	6140000	31600000	13300000	15100000	21600000	5,70E+07	8480000
P01015	209000	27200	566000	688000	774000	287000	3120000	126000	59100	2140000	488000	538000
F1LTN6	11200000	2710000	27700000	1,20E+07	15600000	5410000	18900000	14800000	12500000	10900000	6890000	6710000
M0R9U2	227000	669000	1600000	22400	488000	159000	253000	453000	347000	134000	73600	224000
Q5I0M3	37600000	50800000	62300000	39300000	99400000	7720000	52900000	26200000	65800000	33900000	54700000	18300000
G3V6G1	7730000	7800000	8100000	9680000	9200000	2770000	7300000	3720000	7390000	6940000	6020000	5510000
P08025	183000	NA	226000	46200	2130000	NA	2830000	1230000	1190000	4650000	734000	149000
Q5FWS5	19800000	6970000	16800000	25600000	35800000	1830000	28200000	8940000	1,10E+07	10100000	30600000	4980000
F7FMS0	1070000	804000	1980000	2520000	2930000	127000	3200000	693000	1480000	1890000	1410000	566000
B5DEH7	36500000	65200000	71400000	49700000	63800000	4010000	52300000	23500000	15100000	27700000	6,50E+07	9440000
G3V9J1	21800000	18200000	30600000	23100000	41700000	1250000	11800000	11400000	2670000	6400000	3,90E+07	2300000
Q63556	472000	270000	357000	1460000	1080000	639000	2770000	581000	676000	3120000	358000	1360000
Q4KM75	6810000	11500000	6590000	6590000	7930000	2580000	9080000	2990000	6530000	6130000	4930000	5790000
Q5BK77	6920000	3350000	7460000	5650000	9930000	507000	2680000	708000	2100000	712000	1890000	472000
F1LPW0	1,42E+08	1,61E+08	1,94E+08	1,37E+08	1,73E+08	60600000	1,69E+08	62400000	51500000	71800000	1,46E+08	87300000
G3V9R2	4,98E+08	5,09E+08	5,41E+08	4,33E+08	1,06E+09	1,45E+08	6,57E+08	2,26E+08	3,70E+08	4,70E+08	5,45E+08	2,73E+08
F1LYU4	177000	NA	206000	252000	80100	31100	243000	NA	36200	120000	NA	176000
A0A0G2K7P6	299000	144000	382000	428000	444000	281000	623000	149000	163000	347000	327000	264000
M0R485	42800000	30800000	76300000	56300000	94500000	9160000	66300000	18600000	13700000	24500000	62400000	12400000
Q5FVN3	358000	717000	NA	302000	752000	NA	11300000	1870000	2590000	4480000	8490000	399000
Q05820	834000	1220000	876000	1750000	2390000	193000	2950000	3020000	2570000	2800000	3590000	1200000
A0A0G2JZV7	207000	NA	206000	252000	80100	31100	243000	NA	36200	120000	NA	397000
A0A0G2K975	5,00E+07	19800000	41800000	2,80E+07	65700000	3770000	53800000	19400000	22400000	25100000	3,10E+07	9910000
F1LXY6	1590000	3340000	4640000	1360000	2470000	1150000	1960000	402000	1330000	655000	1940000	1760000
Q7TQ11	23200000	30800000	35200000	21700000	36400000	4790000	15600000	10700000	9530000	1,00E+07	30500000	6260000
Q80ZA3	15100000	19900000	32300000	29900000	41300000	4800000	24500000	10300000	5170000	15400000	29900000	5650000
D3ZPI8	6650000	1,10E+07	9890000	9840000	6950000	864000	9920000	5420000	3090000	7190000	3300000	1820000
F1LZ11	633000	717000	5,00E+05	713000	493000	287000	564000	237000	49300	178000	502000	371000
F1LWS4	6100000	8930000	9790000	5220000	1,20E+07	943000	5980000	3330000	2070000	2770000	9320000	1520000
A0A0G2K7X7	590000	679000	370000	553000	2250000	31200	5120000	192000	518000	1820000	606000	1220000
D3ZWD6	15600000	20700000	19600000	25200000	12500000	1010000	20900000	9940000	9920000	20700000	5590000	1580000
Q5M8C3	2540000	1880000	7890000	2250000	4370000	2020000	4060000	863000	312000	1110000	3580000	1310000
Q5EBA7	1740000	2820000	5590000	690000	4850000	164000	2050000	258000	1120000	352000	4320000	625000
A0A0G2JYC4	977000	1,20E+07	2490000	916000	4300000	176000	1400000	958000	844000	514000	2110000	344000
P68035	3050000	1400000	199000	4890000	NA	2150000	610000	1230000	505000	279000	297000	71400
P68136	3050000	1400000	199000	4890000	NA	2150000	610000	1230000	505000	279000	297000	71400
P62738	3050000	1400000	199000	4890000	NA	2150000	610000	1230000	505000	279000	297000	71400
P63269	3050000	1400000	199000	4890000	NA	2150000	610000	1230000	505000	279000	297000	71400
P63259	4080000	1790000	199000	6,00E+06	NA	2930000	788000	1580000	505000	279000	386000	71400
P60711	4080000	1790000	199000	6,00E+06	NA	2930000	788000	1580000	505000	279000	386000	71400
Q5M7T5	4940000	10700000	2040000	1940000	15500000	205000	3870000	3080000	1590000	5880000	3430000	673000
B1H260	473000	11500000	1300000	824000	1290000	NA	331000	262000	167000	328000	1750000	91700
D3ZQ25	753000	7700000	2170000	1330000	2440000	NA	835000	491000	58200	147000	2780000	32200
Q9EQV9	749000	NA	1050000	686000	1430000	49800	1250000	689000	198000	415000	531000	93100
Q6QMY6	459000	176000	248000	801000	296000	NA	984000	NA	527000	803000	NA	83900

Accession	Log2FC.RC1.T50	Log2FC.RC3.T50	Log2FC.RC4.T50	Log2FC.RC5.T50	Log2FC.RC6.T50	Log2FC.RC7.T50	Log2FC.RL1.T50	Log2FC.RL10.T50	Log2FC.RL11.T50	Log2FC.RL12.T50	Log2FC.RL13.T50	Log2FC.RL9.T50	Description
P20059	5,511516424	-9,391090119	-3,444464279	-3,444464279	-4,457905764	0,817939673	3,778104289	NA	-1,925446415	8,921796272	-0,944236728	-1,300509111	Hemopexin OS=Rattus norvegicus (Rat) OX=10116 GN=Hpx PE=1 SV=3
Q9QX79	3,475127949	-5,537096189	NA	NA	-5,368221747	-2,236536604	0,455271424	NA	-2,236725772	4,744847236	-1,072568626	NA	Fetuin-B OS=Rattus norvegicus (Rat) OX=10116 GN=Fetub PE=2 SV=2
M0RA79	1,933108116	-1,311201688	1,868210127	1,868210127	0,839121968	NA	-0,184839675	-2,309410055	-0,728553354	NA	-0,717269793	1,036876002	Ig-like domain-containing protein OS=Rattus norvegicus (Rat) OX=10116 GN=ENSRNOG00000048425 PE=1 SV=1
M0R816	0,712749357	-1,638460049	0,192645078	0,192645078	-3,118055762	NA	0,99527759	-1,093396536	NA	-0,44012848	-1,181710916	-0,369551227	Ig-like domain-containing protein OS=Rattus norvegicus (Rat) OX=10116 GN=ENSRNOG00000055193 PE=1 SV=2
A0A0G2K0N6	-0,089005006	-1,825669819	NA	NA	-0,021533162	NA	0,645083493	1,700920056	-0,10515823	-1,799840868	-0,566308817	0,936889956	Ig-like domain-containing protein OS=Rattus norvegicus (Rat) OX=10116 GN=ENSRNOG00000057220 PE=1 SV=1
F7FHF3	-0,090602549	-0,428383525	1,599870708	1,599870708	-0,686560641	-2,741983669	1,862042727	0,333423734	-0,283665739	0,591099027	-0,336839804	0,620295574	SERPIN domain-containing protein OS=Rattus norvegicus (Rat) OX=10116 GN=Serpinf2 PE=1 SV=1
P09005	-0,13135324	0,439764585	1,979924223	1,979924223	-0,764238524	1,728479128	2,517955812	-2,074978569	0,747419952	1,80279023	-0,705193248	1,242655325	Serine protease inhibitor 2.1 OS=Rattus norvegicus (Rat) OX=10116 GN=- PE=2 SV=1
D4A817	-0,180432659	-0,525065547	NA	NA	NA	NA	-1,659623506	2,651749592	NA	-1,252387162	NA	0,605721061	Histone H2B OS=Rattus norvegicus (Rat) OX=10116 GN=Hist2h2be PE=3 SV=3
D3ZFH5	-0,201453084	-0,318109756	2,74375476	2,74375476	-1,93986184	-0,368306449	2,343407822	1,082893008	-1,098110085	1,328948523	-0,33824075	1,17362167	Inter-alpha-trypsin inhibitor heavy chain 2 OS=Rattus norvegicus (Rat) OX=10116 GN=Itih2 PE=1 SV=3
G3V8D4	-0,264323102	-0,794190463	0,674904626	0,674904626	0,04608239	-3,229361976	-0,523933067	-2,454320471	-2,257899424	-2,160354463	-0,200232355	1	Apolipoprotein C-II (Predicted) OS=Rattus norvegicus (Rat) OX=10116 GN=Apoc2 PE=1 SV=1
D3ZPL2	-0,316907525	-1,335163922	NA	NA	-1,325670497	NA	NA	NA	1,123599378	1,417017863	NA	1,468808756	Ig-like domain-containing protein OS=Rattus norvegicus (Rat) OX=10116 GN=ENSRNOG00000057548 PE=1 SV=3
Q5GAM5	-0,387730153	1,145086695	1,336386148	1,336386148	0,583199892	-2,633645991	1,809478094	0,989307664	0,952322025	1,070271079	0,333423734	3,525211693	RNAse_Pc domain-containing protein OS=Rattus norvegicus (Rat) OX=10116 GN=Ang2 PE=1 SV=1
Q5EBC0	-0,480625841	-0,72762773	1,084585332	1,084585332	-0,650415562	-2,526747887	0,337996464	-1,811996304	-2,225119634	-0,49777793	-0,0176094	0,465825609	Inter alpha-trypsin inhibitor heavy chain 4 OS=Rattus norvegicus (Rat) OX=10116 GN=Itih4 PE=1 SV=1
P01015	-0,644993215	0,397519738	2,420575683	2,420575683	-1,846479079	NA	3,899971182	2,211745177	-3,259572018	1,637130327	-0,665452419	0,906555436	Angiotensinogen OS=Rattus norvegicus (Rat) OX=10116 GN=Agt PE=1 SV=1
F1LTN6	-0,926560576	-0,947877269	1,074580326	1,074580326	2,781953049	-3,687922971	0,754887502	2,449232419	-1,147957881	-0,138706271	-1,178970141	0,310684172	Uncharacterized protein OS=Rattus norvegicus (Rat) OX=10116 GN=ENSRNOG00000049829 PE=1 SV=2
M0R9U2	-0,9510904	-0,255461186	1,266012106	1,266012106	-1,919829651	NA	0,156445087	-0,562495161	-2,205064337	2,580662363	-2,729103476	0,494471967	Ig-like domain-containing protein OS=Rattus norvegicus (Rat) OX=10116 GN=ENSRNOG00000050708 PE=1 SV=1
Q5I0M3	-0,954459216	-0,1127296	1,204358499	1,204358499	0,549085464	-2,581133182	0,49253506	-0,955261685	0,078855421	-0,213244039	-0,861705019	1,245170896	Complement component factor h-like 1 OS=Rattus norvegicus (Rat) OX=10116 GN=Cfhr1 PE=1 SV=1
G3V6G1	-0,961931959	-0,271168834	1,357010554	1,357010554	-0,14870173	-4,440572591	-0,08257195	-1,068171503	-0,132347544	-0,480071385	-0,611870374	0,992166343	Immunoglobulin joining chain OS=Rattus norvegicus (Rat) OX=10116 GN=Jchain PE=1 SV=1
P08025	-1,044925871	3,491853096	NA	NA	1,981384322	-5,300479676	3,950886499	NA	2,396566896	6,653194054	-1,537001462	NA	Insulin-like growth factor I OS=Rattus norvegicus (Rat) OX=10116 GN=Igf1 PE=1 SV=3
Q5FWS5	-1,114332675	0,98701728	1,924168587	1,924168587	-0,587229108	-2,514941913	0,510194732	0,359116175	-0,610957709	-1,341788517	-0,226427935	1,444302094	Lysyl oxidase-like 1 OS=Rattus norvegicus (Rat) OX=10116 GN=Loxl1 PE=2 SV=1
F7FMS0	-1,145966704	1,324256901	1,712266825	1,712266825	-0,572081209	-3,14059833	1,580461108	-0,214340149	-0,419903254	-0,415037499	-1,055205502	2,155973556	Macrophage-stimulating 1 OS=Rattus norvegicus (Rat) OX=10116 GN=Mst1 PE=3 SV=1
B5DEH7	-1,186107015	0,19730144	1,317029295	1,317029295	0,473665229	-2,525461489	0,518914482	-1,472211208	-2,241375525	-0,843359876	0,026883294	1,235184623	C1r protein OS=Rattus norvegicus (Rat) OX=10116 GN=C1r PE=1 SV=1
G3V9J1	-1,215337664	-0,905593791	0,334984248	0,334984248	0,285621091	-5,175923742	-0,885541275	-0,674904626	-3,518620006	-1,851749041	-0,09657326	0,879705766	Uncharacterized protein OS=Rattus norvegicus (Rat) OX=10116 GN=ENSRNOG00000042228 PE=1 SV=3
Q63556	-1,224145392	0,555762524	2,247927513	2,247927513	-0,140007479	-0,309955453	2,553027212	1,105578756	0,921099172	1,09557766	-1,59299982	1,089718815	Serine protease inhibitor A3M OS=Rattus norvegicus (Rat) OX=10116 GN=Serpina3m PE=2 SV=1
Q4KM75	-1,229970614	-0,156796105	2,112639261	2,112639261	0,49704591	-4,447583299	0,415037499	-1,943416472	-0,013195473	-0,104391391	-0,685733219	1,166192283	CD5 antigen-like OS=Rattus norvegicus (Rat) OX=10116 GN=Cd5l PE=1 SV=1
Q5BK77	-1,245270126	0,913508474	1,147845468	1,147845468	0,429784085	-2,196217784	-1,368539037	-2,24233983	-1,828786303	-2,988301721	-2,393407483	-0,103198888	Retinoic acid receptor responder (Tazarotene induced) 2 OS=Rattus norvegicus (Rat) OX=10116 GN=Rarres2 PE=1 SV=1
F1LPW0	-1,331367791	0,04580369	1,367277082	1,367277082	-0,37077958	-1,899356923	0,251132317	-1,367442754	-1,913412315	-0,932120144	-0,244803669	0,52666386	Immunoglobulin heavy constant mu OS=Rattus norvegicus (Rat) OX=10116 GN=Ighm PE=1 SV=1
G3V9R2	-1,369668684	-0,920565533	1,331357508	1,331357508	0,045442971	-2,095562399	0,399747628	-1,171342884	-0,548103323	0,118293732	-0,95973613	0,912848051	Complement factor H OS=Rattus norvegicus (Rat) OX=10116 GN=Cfh PE=1 SV=1
F1LYU4	-1,382616022	-0,176098769	2,628031223	2,628031223	NA	NA	0,457206954	NA	-2,508582735	-1,070389328	NA	2,500588943	Ig-like domain-containing protein OS=Rattus norvegicus (Rat) OX=10116 GN=ENSRNOG00000049009 PE=4 SV=3
A0A0G2K7P6	-1,398549376	NA	Inf	Inf	-0,664076761	-1,148445274	1,059086679	0,049243519	-1,228700674	-0,302675134	-0,441269041	-0,090032201	IGv domain-containing protein OS=Rattus norvegicus (Rat) OX=10116 GN=ENSRNOG00000057376 PE=4 SV=1
M0R485	-1,418254212	0,691877705	1,264827688	1,264827688	-0,026472211	-2,021268617	0,631398074	-0,72762773	-2,477507164	-1,200353173	-0,5987683	0,436920617	PGRP domain-containing protein OS=Rattus norvegicus (Rat) OX=10116 GN=Pglyrp2 PE=1 SV=2
Q5FVN3	-1,438763569	NA	NA	NA	1,123792486	NA	4,980219375	1,382993246	NA	3,890878278	3,496959987	NA	SCY domain-containing protein OS=Rattus norvegicus (Rat) OX=10116 GN=Ccl9 PE=1 SV=1
Q05820	-1,451988635	0,66792395	2,426036498	2,426036498	-0,873970347	-3,285247432	1,822595666	1,307667402	1,552765584	0,678071905	0,586973226	2,636361653	Putative lysozyme C-2 OS=Rattus norvegicus (Rat) OX=10116 GN=Lyz2 PE=5 SV=1
A0A0G2JZV7	-1,45845978	-0,176098769	2,628031223	2,628031223	NA	NA	0,231325546	NA	-2,508582735	-1,070389328	NA	3,674152522	Ig-like domain-containing protein OS=Rattus norvegicus (Rat) OX=10116 GN=ENSRNOG00000047464 PE=1 SV=1
A0A0G2K975	-1,556833128	-1,238347527	1,294447358	1,294447358	-0,921455223	-1,360500215	0,105678078	-0,029443778	-0,90000421	-0,157739463	-1,083625155	1,394320534	Similar to complement factor H-related protein OS=Rattus norvegicus (Rat) OX=10116 GN=RGD1564614 PE=1 SV=1
F1LXY6	-1,742420024	-0,79113457	2,174497731	2,174497731	-0,505374236	NA	0,301826889	-3,054580696	-1,80269856	-1,05403984	-0,348454389	0,613941568	Ig-like domain-containing protein OS=Rattus norvegicus (Rat) OX=10116 GN=ENSRNOG00000058824 PE=1 SV=2
Q7TQ11	-1,82944651	-0,62298884	1,351354209	1,351354209	-0,830369156	-2,992359938	-0,572578776	-1,525319554	-1,88502731	-1,117695043	-0,255129208	0,386137001	SMB domain-containing protein OS=Rattus norvegicus (Rat) OX=10116 GN=Vtn PE=1 SV=1
Q80ZA3	-1,830600666	0,980079306	1,631203532	1,631203532	0,256564911	-1,831318165	0,6982332	-0,950124093	-2,643297979	-0,957215134	-0,465996297	0,235216462	SERPIN domain-containing protein OS=Rattus norvegicus (Rat) OX=10116 GN=Serpinf1 PE=1 SV=1
D3ZPI8	-1,915572839	-0,324272035	1,337248894	1,337248894	-0,263034406	-2,049147649	0,57698578	-1,021138767	-1,678363683	-0,452666545	-1,074546953	1,074835233	Lipocln_cytosolic_FA-bd_dom domain-containing protein OS=Rattus norvegicus (Rat) OX=10116 GN=C8g PE=1 SV=1
F1LZ11	-2,099535674	-0,484746471	2,9777393	2,9777393	NA	NA	-0,166510337	-1,59708606	-3,342268543	-2,002024835	0,026099718	0,37036845	Ig-like domain-containing protein OS=Rattus norvegicus (Rat) OX=10116 GN=ENSRNOG00000042319 PE=1 SV=1
F1LWS4	-2,393738097	-1,144543936	1,207892852	1,207892852	-0,773197911	-2,714458195	-0,028663758	-1,423137998	-2,241678092	-0,914163831	-0,364632546	0,688741648	Uncharacterized protein OS=Rattus norvegicus (Rat) OX=10116 GN=Cfhr2 PE=1 SV=3
A0A0G2K7X7	-2,446971498	1,166410517	3,20278083	3,20278083	1,421826665	-0,216198854	3,117356951	-1,822305264	0,485426827	1,718587065	-1,892535303	5,289191308	Complement C7 OS=Rattus norvegicus (Rat) OX=10116 GN=C7 PE=1 SV=1
D3ZWD6	-2,662352533	-0,191048293	1,661238268	1,661238268	-0,044891457	-3,484075746	0,421956913	-1,058313011	-0,982441629	-0,283792966	-1,161007907	0,645569265	MACPF domain-containing protein OS=Rattus norvegicus (Rat) OX=10116 GN=C8a PE=1 SV=1
Q5M8C3	-2,97016477	-0,426264755	2,87782813	2,87782813	-2,225881407	1,082589781	0,67665123	-1,123300197	-4,660407366	-1,019365325	-0,287673692	-0,624788481	SERPIN domain-containing protein OS=Rattus norvegicus (Rat) OX=10116 GN=Serpina4 PE=1 SV=1
Q5EBA7	-2,999182841	-0,396283539	2,459787006	2,459787006	-1,253756592	NA	0,236536604	-3,450252192	-2,319349551	-0,971020933	-0,166953435	1,930160375	HGF activator OS=Rattus norvegicus (Rat) OX=10116 GN=Hgfac PE=1 SV=1
A0A0G2JYC4	-3,09838474	-0,926279581	1,196397213	1,196397213	-0,55987152	-4,457732831	0,51899636	-3,64686494	-1,560830838	-0,833579239	-1,027093661	0,966833136	Complement factor H-related protein B OS=Rattus norvegicus (Rat) OX=10116 GN=LOC100361907 PE=1 SV=1
P68035	-3,223929656	1,274174963	NA	NA	-5,812127672	0,257909374	-2,321928095	-0,186768512	1,343514957	-4,131497438	NA	-4,912268775	Actin alpha cardiac muscle 1 OS=Rattus norvegicus (Rat) OX=10116 GN=Actc1 PE=2 SV=1
P68136	-3,223929656	1,274174963	NA	NA	-5,812127672	0,257909374	-2,321928095	-0,186768512	1,343514957	-4,131497438	NA	-4,912268775	Actin alpha skeletal muscle OS=Rattus norvegicus (Rat) OX=10116 GN=Acta1 PE=1 SV=1
P62738	-3,223929656	1,274174963	NA	NA	-5,812127672	0,257909374	-2,321928095	-0,186768512	1,343514957	-4,131497438	NA	-4,912268775	Actin aortic smooth muscle OS=Rattus norvegicus (Rat) OX=10116 GN=Acta2 PE=2 SV=1
P63269	-3,223929656	1,274174963	NA	NA	-5,812127672	0,257909374	-2,321928095	-0,186768512	1,343514957	-4,131497438	NA	-4,912268775	Actin gamma-enteric smooth muscle OS=Rattus norvegicus (Rat) OX=10116 GN=Actg2 PE=2 SV=1
P63259	-3,395928676	0,950389552	NA	NA	-6,066311161	0,257909374	-2,372301617	-0,180035029	1,343514957	-4,426625474	NA	-5,35883278	Actin cytoplasmic 2 OS=Rattus norvegicus (Rat) OX=10116 GN=Actg1 PE=1 SV=1
P60711	-3,395928676	0,950389552	NA	NA	-6,066311161	0,257909374	-2,372301617	-0,180035029	1,343514957	-4,426625474	NA	-5,35883278	Actin cytoplasmic 1 OS=Rattus norvegicus (Rat) OX=10116 GN=Actb PE=1 SV=1
Q5M7T5	-3,751436077	-3,228335295	-0,156455455	-0,156455455	-3,177538186	-3,12529877	-0,352177475	-1,796608541	-0,359542387	1,599759503	-2,175987734	1,714982595	SERPIN domain-containing protein OS=Rattus norvegicus (Rat) OX=10116 GN=Serpinc1 PE=1 SV=1
B1H260	NA	-0,851901361	NA	NA	#NOM?	NA	-0,515008967	-5,455923239	-2,960591615	-1,328948523	0,439983856	NA	Coagulation factor XIII B chain OS=Rattus norvegicus (Rat) OX=10116 GN=F13b PE=1 SV=1
D3ZQ25	NA	-0,502500341	1,553153762	1,553153762	0,206253775	NA	0,149126333	-3,971063516	-5,220532079	-3,177538186	0,188203735	NA	Fibulin-1 OS=Rattus norvegicus (Rat) OX=10116 GN=Fbln1 PE=1 SV=1
Q9EQV9	NA	0,779963	1,59917636	1,59917636	-2,01774555	NA	0,738890471	NA	-2,406816992	-0,72509724	-1,429231381	0,902635425	Carboxypeptidase B2 OS=Rattus norvegicus (Rat) OX=10116 GN=Cpb2 PE=2 SV=1
Q6QMY6	NA	2.193603998	NA	NA	NA	NA	1.100164162	NA	1.087462841	0.003597745	NA	NA	Tsukushin OS=Rattus norvegicus (Rat) OX=10116 GN=Tsku PE=2 SV=1

Supplemental Table 3: Identifications and calculation of $Fold_2Change$ of proteins specific to T50 groups obtained from LC-MS/MS of control and endotoxemic shock rat plasma samples.
NA corresponds to missing values.

Accession	Peptide Unique	Coverage	Area.C90.T0.2
Q6MG90	41	80	167000
D3ZQ25	30	38	160000
A0A0G2JXI1	40	23	44900
Q8CHN8	43	53	226000
P31394	12	33	28300
B1H260	42	56	169000
P18420	15	75	27800
Q5M890	22	64	508000
Q63313	25	49	51400
P16296	11	24	137000
Q5M8C3	23	55	137000
B5DEH7	94	82	7,00E+06
M0R816	8	44	38100
Q4KM75	18	47	428000
Q5EBA7	19	26	125000
A0A0G2JYC4	25	58	632000
M0RA79	5	60	103000
Q5FWS5	33	52	1030000
Q63207	12	29	121000
Q7TQ11	28	34	2220000
Q5EBC0	113	84	6880000
F1LPW0	69	63	12900000
F1LWS4	36	62	1280000
Q5FVN3	13	68	65900
Q80ZA3	55	74	2700000
F1LTN6	22	70	2220000
P58775	4	35	404000
G3V8D4	47	90	55700000
D3ZPI8	15	86	1320000
D3ZWD6	55	78	2450000
G3V6G1	13	56	791000
D3ZFH5	17	23	84600
Q5GAM5	15	61	868000
M0R485	44	69	4780000
Q5I0M3	39	89	6840000
G3V9R2	265	93	80400000
F7FMS0	19	29	168000
A0A0G2K975	75	76	5780000
F7FHF3	12	20	303000
F1LTY5	2	28	61500
G3V9J1	36	68	4260000
Q5BK77	15	61	762000
Q5M7T5	38	54	1920000
O08619	43	46	1080000
A0A0G2K7X7	15	20	61000
P08649	11	86	NA
Q64240	20	42	NA
D4ACR1	8	83	NA
F1LXY6	11	100	NA

F1LZ11	6	55	NA
P07151	8	81	NA
M0R9U2	4	39	NA
M0R4C5	4	30	NA

Area.C90.T0.3	Area.C90.T0.4	Area.C90.T0.5
356000	1310000	3430000
412000	757000	1650000
115000	47300	701000
1220000	553000	3560000
230000	53300	329000
422000	2250000	1600000
96000	105000	352000
3580000	4570000	4380000
251000	179000	415000
898000	654000	1460000
114000	57600	725000
10500000	9130000	32300000
NA	34000	208000
770000	2110000	4800000
86600	452000	926000
322000	1680000	983000
NA	NA	253000
1030000	371000	2140000
116000	121000	717000
2630000	4950000	11700000
6250000	7420000	18600000
19300000	2,50E+07	1,04E+08
1340000	1340000	3390000
27400	38400	18400
2380000	3210000	6440000
4670000	NA	30100000
93300	NA	512000
1,00E+08	1,28E+08	1,93E+08
1100000	1740000	4260000
1030000	2590000	7420000
393000	1250000	3920000
31800	NA	154000
1020000	343000	1560000
6860000	4420000	15400000
7840000	9450000	18200000
85400000	97900000	2,24E+08
137000	91900	272000
4330000	2030000	10400000
156000	20500	183000
NA	NA	415000
4110000	2690000	10100000
457000	221000	1540000
297000	2130000	1580000
NA	79300	297000
NA	35000	NA
48300	415000	3390000
73300	241000	298000
25800	61100	661000
69000	363000	2750000

	27900	43300	612000
	60300	207000	401000
NA		42400	159000
NA		88700	266000

Area.C90.T0.7	Area.C90.T0.8	Area.C90.T90.2
21300000	23400	23700000
1,20E+07	74600	16400000
4480000	118000	4320000
14300000	NA	21400000
2620000	848000	2070000
11500000	NA	11900000
1950000	NA	1930000
54900000	NA	2,40E+07
4810000	NA	1770000
6600000	137000	4650000
2590000	255000	4270000
80600000	14600000	1,98E+08
2060000	NA	987000
29500000	3160000	10700000
3210000	245000	3090000
8650000	349000	12500000
1520000	79500	2020000
14200000	11300000	19800000
2970000	733000	2020000
48900000	13400000	35800000
5,30E+07	6610000	1,00E+08
5,04E+08	35900000	1,73E+08
19300000	1460000	1,60E+07
150000	253000	768000
1,80E+07	6710000	30500000
1,26E+08	12600000	22600000
8,00E+05	77400	3900000
7,67E+08	70300000	5,29E+08
11200000	3880000	12500000
25400000	1,20E+07	22700000
20300000	4020000	7160000
593000	920000	712000
9200000	5120000	7190000
33700000	23100000	39500000
70900000	28600000	52800000
6,94E+08	2,73E+08	6,20E+08
832000	1310000	1240000
43500000	23100000	42100000
3170000	1530000	2200000
530000	220000	369000
23200000	3120000	22600000
4140000	3820000	3630000
6320000	2790000	8710000
5,00E+06	NA	4770000
186000	259000	159000
13800000	NA	22600000
2230000	8470	10600000
278000	NA	341000
4490000	377000	1100000

	2050000	135000	966000
NA		26100	1140000
	1430000	246000	861000
	934000	206000	1290000

Area.C90.T90.3	Area.C90.T90.4	Area.C90.T90.5
1080000	1410000	NA
468000	1210000	86900
13700	4040000	23800
501000	8670000	236000
87900	2380000	175000
411000	1160000	106000
24700	426000	NA
2280000	448000	41800
32300	1540000	NA
170000	4120000	779000
618000	4550000	NA
6810000	65400000	15200000
30800	139000	91300
407000	11300000	6210000
121000	7780000	169000
252000	5810000	697000
NA	244000	374000
726000	13400000	2800000
62700	4320000	1030000
7480000	1,50E+07	2420000
3,30E+07	52200000	11500000
17900000	1,68E+08	43700000
456000	13200000	1620000
NA	7050000	1970000
1060000	3,50E+07	1430000
3600000	NA	1,80E+07
NA	540000	52600
4,70E+07	1,85E+08	76700000
1080000	14200000	2200000
1010000	36300000	5090000
447000	10400000	5340000
256000	2870000	9940
70400	22200000	6870000
1520000	63100000	5470000
4350000	9,60E+07	29100000
50200000	7,76E+08	1,98E+08
74900	4600000	542000
1640000	56300000	12500000
114000	7250000	1200000
7650	726000	575000
1040000	18400000	7340000
26500	9060000	3630000
831000	4080000	1020000
169000	NA	NA
NA	961000	NA
280000	831000	2,00E+05
245000	494000	NA
NA	988000	170000
178000	2450000	1090000

	31900	369000	111000
	33500	678000	149000
	41400	224000	51300
NA		239000	0

Area.C90.T90.7	Area.C90.T90.8	Area.L90.T0.2
7560000	NA	7320000
5920000	NA	8230000
3640000	NA	3160000
1,50E+07	NA	13900000
3200000	27300	2550000
9030000	NA	7270000
1060000	NA	721000
21900000	NA	12800000
3120000	NA	1210000
4690000	NA	1450000
2560000	NA	2150000
95100000	1940000	75800000
1300000	NA	953000
24200000	539000	7020000
4050000	NA	1680000
7890000	NA	2020000
743000	NA	335000
12200000	1580000	1,40E+07
2670000	76000	1160000
35900000	665000	43200000
75100000	626000	31100000
3,67E+08	8320000	1,27E+08
27400000	NA	11700000
585000	61900	NA
18400000	441000	16100000
1,04E+08	1130000	26700000
728000	NA	2450000
6,88E+08	9980000	6,47E+08
8930000	395000	5290000
19800000	411000	11500000
16600000	1950000	6300000
517000	NA	533000
14100000	2250000	3360000
30100000	1770000	30400000
73500000	2410000	30100000
6,41E+08	2,80E+07	3,20E+08
1080000	NA	468000
55700000	2890000	20300000
3810000	NA	2630000
330000	19700	415000
34400000	52900	20700000
1140000	1220000	1390000
5590000	135000	3390000
1390000	91300	2640000
94000	15300	34500
6970000	NA	2480000
2680000	NA	1510000
194000	NA	NA
3220000	NA	2140000

	1880000	NA		435000
NA		NA		102000
	854000	NA	NA	
	856000	NA		584000

Area.L90.T0.3	Area.L90.T0.5	Area.L90.T0.6
26500000	17100000	42300000
1,60E+07	21300000	16300000
6650000	12900000	3930000
9280000	29600000	16400000
1250000	3280000	1180000
8850000	2,40E+07	12200000
634000	1090000	3310000
86800000	1,60E+08	6,50E+07
6090000	8960000	1660000
1800000	3070000	4820000
7740000	28700000	4820000
1,01E+08	1,33E+08	1,47E+08
69900	883000	1030000
9250000	12400000	17100000
2680000	10100000	5310000
7560000	22600000	6,00E+06
619000	1040000	1110000
10200000	22100000	9850000
2540000	3790000	1980000
21400000	53700000	33900000
43500000	1,67E+08	73600000
2,35E+08	3,16E+08	3,18E+08
14400000	30800000	9240000
103000	834000	202000
25100000	74900000	35800000
29400000	69800000	1,12E+08
1050000	1540000	202000
3,81E+08	1,17E+09	8,26E+08
14500000	2,50E+07	6620000
2,20E+07	41200000	8300000
9580000	1,40E+07	9590000
212000	827000	739000
1650000	10400000	3450000
27400000	91500000	60700000
2,20E+07	95600000	52900000
5,03E+08	1,14E+09	7,62E+08
1020000	4340000	653000
21700000	52900000	20100000
1070000	3350000	1570000
430000	917000	545000
20400000	57900000	23800000
4250000	7390000	3610000
25600000	7760000	4900000
15800000	9930000	14700000
125000	1180000	179000
7870000	3200000	23500000
8240000	13300000	5180000
1410000	2850000	1480000
7,00E+06	7780000	1090000

1330000	3670000	565000
170000	1640000	2390000
672000	1180000	1960000
749000	880000	2330000

Area.L90.T0.7	Area.L90.T0.8	Area.L90.T90.2
2,00E+07	5350000	740000
14100000	2630000	1310000
8110000	903000	931000
17300000	4800000	3400000
2360000	1030000	1430000
10200000	3390000	5,00E+05
1770000	NA	202000
15500000	30300000	540000
9080000	1890000	1390000
4240000	1350000	999000
4990000	3580000	694000
1,85E+08	36200000	42400000
1740000	146000	4,00E+05
13100000	4030000	4720000
4730000	1810000	1500000
11500000	3010000	497000
24900	15000	281000
17200000	3150000	25800000
2980000	384000	1470000
65300000	14300000	10600000
6,27E+08	34100000	4,00E+07
1,70E+08	91400000	72300000
9950000	7150000	3830000
334000	207000	1300000
20100000	12200000	18900000
95900000	27900000	8660000
3080000	2040000	1,00E+07
5,33E+08	2,97E+08	2,58E+08
1,40E+07	4800000	3570000
30300000	6680000	10300000
8070000	2590000	4920000
4450000	389000	1600000
4610000	3330000	8430000
36900000	25200000	34200000
73100000	21200000	3,50E+07
9,23E+08	3,18E+08	1,36E+08
1710000	470000	1960000
65600000	16300000	16100000
4500000	739000	6410000
279000	107000	712000
13600000	15500000	16600000
4780000	1320000	869000
20400000	2570000	427000
3650000	279000	12700
960000	NA	839000
20800000	1140000	249000
5490000	2630000	241000
NA	63600	155000
635000	1360000	1560000

482000	426000		154000
8420000	324000	NA	
1330000	657000		171000
341000	59800		224000

Area.L90.T90.3	Area.L90.T90.5	Area.L90.T90.6
3910000	1040000	15700000
5540000	1770000	6870000
1160000	3320000	3670000
14400000	10900000	12600000
1930000	2220000	308000
4200000	937000	6220000
1470000	534000	2510000
4630000	3270000	23600000
3820000	2,00E+06	4370000
2540000	1780000	4290000
3240000	4250000	1640000
1,22E+08	67600000	97400000
330000	593000	627000
18200000	9500000	7700000
2550000	3660000	4080000
5040000	3170000	4560000
287000	874000	352000
27800000	19400000	8490000
1,00E+06	2080000	1390000
3,10E+07	20600000	27300000
70300000	48300000	56900000
3,52E+08	1,62E+08	1,68E+08
12200000	12300000	7100000
1240000	575000	298000
41600000	27900000	12100000
1,04E+08	82300000	47700000
1540000	811000	819000
3,20E+08	3,50E+08	4,04E+08
13800000	9410000	2750000
30100000	20600000	2470000
16800000	10700000	5900000
3010000	2550000	343000
18300000	15900000	5070000
55400000	56400000	37800000
5,50E+07	1,00E+08	32500000
8,38E+08	7,08E+08	4,76E+08
1310000	1810000	372000
66300000	46800000	1,30E+07
4370000	5880000	1120000
1200000	1020000	305000
52500000	31300000	21500000
1150000	2600000	306000
14600000	3570000	3140000
560000	26300	4110000
966000	979000	401000
4950000	405000	12100000
904000	385000	2430000
1080000	1,30E+07	1380000
4590000	2350000	761000

763000	457000	282000
597000	655000	746000
1100000	913000	1020000
1420000	935000	1140000

Area.L90.T90.7	Area.L90.T90.8	Log2FC.RC2.T90
173000	607000	7,148895146
270000	618000	6,6794801
426000	792000	6,588172057
2580000	3,00E+06	6,565144214
676000	1260000	6,192684904
292000	704000	6,137794517
103000	179000	6,117372154
355000	1260000	5,562062099
1130000	775000	5,105837191
1240000	1530000	5,084982918
908000	1660000	4,961988272
50800000	31600000	4,822001698
445000	228000	4,695187182
4650000	5630000	4,64385619
1790000	1890000	4,627606838
1770000	1760000	4,305859726
NA	187000	4,29363905
11800000	9460000	4,264784188
1600000	1510000	4,06127634
5860000	8160000	4,011328006
40600000	30400000	3,861447625
7,20E+07	97400000	3,745329067
4640000	5020000	3,64385619
7340000	2310000	3,542755941
8880000	11600000	3,49777793
38100000	43800000	3,347691191
5280000	3130000	3,271046926
87100000	1,96E+08	3,24751849
4610000	5730000	3,24331826
9960000	11600000	3,211838643
5220000	5100000	3,178209988
3510000	1810000	3,073147673
15100000	12200000	3,050224823
20800000	31300000	3,04677013
43900000	41100000	2,948469699
4,92E+08	4,31E+08	2,947000809
1500000	878000	2,883806982
38100000	26300000	2,864678835
3350000	3320000	2,860113825
NA	196000	2,584962501
6210000	14800000	2,407397437
890000	1160000	2,252106645
4050000	2690000	2,181566408
NA	NA	2,142957954
455000	122000	1,382145618
859000	507000	NA
231000	262000	NA
NA	672000	NA
212000	6,00E+05	NA

40700	129000	NA
1630000	128000	NA
249000	1020000	NA
1280000	199000	NA

Log2FC.RC3.T90	Log2FC.RC4.T90	Log2FC.RC5.T90
1,601082166	0,106128351	NA
0,183864192	0,676641842	-4,246966037
-3,069386063	6,416371299	-4,880380966
-1,283998639	3,970680608	-3,915018476
-1,387698791	5,48068223	-0,910732662
-0,038104605	-0,955800196	-3,915935735
-1,958523364	2,020464103	NA
-0,650925763	-3,350623528	-6,711284117
-2,958081294	3,104898858	NA
-2,401180699	2,655281797	-0,906273136
2,438573014	6,303653923	NA
-0,624662625	2,84060387	-1,087462841
NA	2,031478231	-1,187896763
-0,919829651	2,421007869	0,371558863
0,482568117	4,105375477	-2,453988947
-0,353636955	1,790076931	-0,49603276
NA	NA	0,563900885
-0,504602884	5,174670004	0,387816031
-0,887587457	5,15795236	0,522599313
1,507975471	1,59946207	-2,273429577
2,40053793	2,814558715	-0,69366876
-0,10864126	2,748461233	-1,250878344
-1,555127271	3,300233024	-1,06529146
NA	7,520373136	6,742346053
-1,166897309	3,44670972	-2,171045541
-0,375425643	NA	-0,74176658
NA	NA	-3,283009106
-1,089267338	0,531381461	-1,331302365
-0,026472211	3,028731719	-0,953349907
-0,028289044	3,808945545	-0,543753531
0,185745519	3,056583528	0,445986088
3,00904514	NA	-3,953540689
-3,856849913	6,01620729	2,13876407
-2,174137253	3,83552173	-1,493317613
-0,849838253	3,344648171	0,677080703
-0,766548706	2,986675887	-0,177998302
-0,871138269	5,645425189	0,9946862
-1,40067121	4,79358329	0,265344567
-0,452512205	8,466213275	2,713118852
NA	NA	0,47045062
-1,982554866	2,774027688	-0,460503325
-4,1081299	5,357392776	1,237039197
1,484385546	0,937715722	-0,631355406
NA	NA	NA
NA	4,779109604	NA
2,535331733	1,001737141	-4,083213368
1,740896646	1,035477895	NA
NA	4,015266757	-1,959115525
1,367208974	2,754740296	-1,335103484

	0,193291302	3,091181886	-2,462971976
	-0,847996907	1,711654506	-1,428289906
NA		2,401362562	-1,631996035
NA		1,430004609	#NOM?

Log2FC.RC7.T90	Log2FC.RC8.T90	Log2FC.RL2.T90
-1,494395291	NA	-3,306246473
-1,019365325	NA	-2,651325619
-0,299560282	NA	-1,763071486
0,068947354	NA	-2,031478231
0,288505093	-4,957091409	-0,8344821
-0,348835968	NA	-3,861955364
-0,879409859	NA	-1,835643966
-1,325875279	NA	-4,567040593
-0,624490865	NA	0,200077835
-0,492878102	NA	-0,537496317
-0,016808288	NA	-1,631329092
0,238665502	-2,911839812	-0,838133584
-0,664132714	NA	-1,252476214
-0,285707907	-2,55156738	-0,572684171
0,335348611	NA	-0,163498732
-0,132674833	NA	-2,023037536
-1,032637208	NA	-0,253590965
-0,219009782	-2,838326309	0,881944238
-0,153623189	-3,269741875	0,34169135
-0,445850621	-4,33273485	-2,026967048
0,502820548	-3,400415709	0,36308542
-0,457643671	-2,109328411	-0,812760945
0,505575046	NA	-1,611092233
1,963474124	-2,03112607	NA
0,03170886	-3,927462206	0,231325546
-0,276840205	-3,479029056	-1,624400812
-0,13606155	NA	2,029146346
-0,156818013	-2,816412969	-1,326394647
-0,326766652	-3,296132094	-0,567343648
-0,359328067	-4,867752202	-0,158989524
-0,290296486	-1,043721377	-0,356693513
-0,197867824	NA	1,585864467
0,615989396	-1,186218809	1,327071398
-0,162985104	-3,706071586	0,169925001
0,051958622	-3,568910095	0,217591435
-0,114611306	-3,285402219	-1,234465254
0,376375879	NA	2,066273219
0,356661927	-2,998751454	-0,334419039
0,265308157	NA	1,285261557
-0,683526335	-3,481235989	0,778765905
0,56828376	-5,882134497	-0,318447526
-1,860596943	-1,64669149	-0,677656801
-0,177076275	-4,36923381	-2,988977298
-1,846843212	NA	-7,699565622
-0,984569959	-4,08134854	4,604002544
-0,985437706	NA	-3,316122473
0,265189291	NA	-2,647443498
-0,519028231	NA	NA
-0,479654757	NA	-0,456064768

	-0,124891248	NA		-1,49808505
NA		NA	NA	
	-0,743707172	NA	NA	
	-0,125811753	NA		-1,382469637

Log2FC.RL3.T90	Log2FC.RL5.T90	Log2FC.RL6T90
-2,760751847	-4,039340892	-1,429893104
-1,530114024	-3,589032165	-1,24648996
-2,519229535	-1,958115919	-0,098749249
0,633872101	-1,441269041	-0,380272081
0,626672753	-0,563136138	-1,937784604
-1,075288127	-4,678841548	-0,971894662
1,21326141	-1,029416488	-0,399143853
-4,228610944	-5,612637459	-1,461652859
-0,67286959	-2,163498732	1,396450038
0,49683159	-0,786361414	-0,168055499
-1,256339753	-2,755515991	-1,555337332
0,272525855	-0,976331094	-0,593822478
2,239101664	-0,574381333	-0,716106989
0,97641318	-0,384340702	-1,151065974
-0,071735754	-1,464439739	-0,380142709
-0,584962501	-2,833768027	-0,395928676
-1,108888673	-0,250878344	-1,656912343
1,446515731	-0,187989717	-0,214359171
-1,344828497	-0,86561432	-0,510415547
0,534657419	-1,382277751	-0,312384322
0,692509288	-1,789753009	-0,371277114
0,582914672	-0,963930745	-0,920565533
-0,239187664	-1,324272035	-0,380073827
3,589623878	-0,536485428	0,560957038
0,728896164	-1,424700597	-1,56495254
1,822695468	0,237665394	-1,231437561
0,552541023	-0,925156531	2,019508159
-0,251719093	-1,741081703	-1,031786489
-0,071384633	-1,409661467	-1,267399598
0,452259963	-1	-1,748600295
0,810363672	-0,387816031	-0,700815861
3,827627317	1,624538013	-1,107365788
3,471305719	0,612443237	0,555389385
1,015710083	-0,698076581	-0,683310282
1,321928095	0,064917477	-0,702828004
0,736391844	-0,687212559	-0,678829424
0,36099766	-1,261705345	-0,81178037
1,611313828	-0,176759193	-0,628683878
2,030022483	0,811655059	-0,487265827
1,480625841	0,153575513	-0,837446987
1,363748271	-0,887400691	-0,146624914
-1,88582898	-1,507062741	-3,560395279
-0,810175441	-1,120132578	-0,64201719
-4,818353921	-8,560587108	-1,838605856
2,950095094	-0,269406095	1,163642649
-0,668935111	-2,982078092	-0,957653709
-3,18824966	-5,11042399	-1,091995784
-0,38466385	2,189477799	-0,100928909
-0,608860768	-1,727109398	-0,518359776

-0,801671284	-3,005513993	-1,002555705
1,812196185	-1,324129003	-1,679763083
0,710970386	-0,370100094	-0,942284502
0,922853306	0,087462841	-1,03129613

Log2FC.RL7.T90	Log2FC.RL8.T90
-6,853084152	-3,13977047
-5,706591945	-2,089384056
-4,250776579	-0,189225557
-2,745329067	-0,678071905
-1,803691708	0,290779396
-5,126456973	-2,267637939
-4,103033118	NA
-5,448305381	-4,587822155
-3,006369525	-1,286118019
-1,773724144	0,180572246
-2,458275613	-1,108776346
-1,864624869	-0,196065139
-1,967210065	0,643065455
-1,49426419	0,482355084
-1,401880596	0,062396537
-2,699812596	-0,774188058
NA	3,640003864
-0,543621705	1,586488355
-0,897240426	1,975370333
-3,478110422	-0,80937409
-3,948913811	-0,165700416
-1,239465935	0,091727607
-1,10057172	-0,510255878
4,457860055	3,480190179
-1,17856392	-0,072756342
-1,331739818	0,650665748
0,777607579	0,617593505
-2,613390909	-0,599609277
-1,602588171	0,255500733
-1,605100146	0,796204798
-0,628518867	0,977545149
-0,342334306	2,218147637
1,711709894	1,873287065
-0,827037288	0,312738923
-0,735650466	0,955074129
-0,907672332	0,438661104
-0,189033824	0,901560183
-0,783904817	0,690190835
-0,425763906	2,167536972
NA	0,873242858
-1,130941478	-0,06667104
-2,425133377	-0,186413124
-2,332575339	0,065837813
NA	NA
-1,077167861	NA
-4,597781587	-1,168976172
-4,570841392	-3,327424083
NA	3,401362562
-1,582692327	-1,180572246

-3,565932447	-1,723482365
-2,368948269	-1,339850003
-2,417208598	0,634603877
1,908300166	1,734551041

Description
Complement C4B (Chido blood group) OS=Rattus norvegicus (Rat) OX=10116 GN=C4b PE=1 SV=1
Fibulin-1 OS=Rattus norvegicus (Rat) OX=10116 GN=Fbln1 PE=1 SV=1
F5/8 type C domain-containing protein OS=Rattus norvegicus (Rat) OX=10116 GN=F5 PE=1 SV=1
Mannan-binding lectin serine protease 1 OS=Rattus norvegicus (Rat) OX=10116 GN=Masp1 PE=1 SV=2
Vitamin K-dependent protein C OS=Rattus norvegicus (Rat) OX=10116 GN=Proc PE=2 SV=1
Coagulation factor XIII B chain OS=Rattus norvegicus (Rat) OX=10116 GN=F13b PE=1 SV=1
Proteasome subunit alpha type-1 OS=Rattus norvegicus (Rat) OX=10116 GN=Psma1 PE=1 SV=2
Apolipoprotein N OS=Rattus norvegicus (Rat) OX=10116 GN=Apon PE=1 SV=1
Lipopolysaccharide-binding protein OS=Rattus norvegicus (Rat) OX=10116 GN=Lbp PE=2 SV=1
Coagulation factor IX OS=Rattus norvegicus (Rat) OX=10116 GN=F9 PE=2 SV=2
SERPIN domain-containing protein OS=Rattus norvegicus (Rat) OX=10116 GN=Serpina4 PE=1 SV=1
C1r protein OS=Rattus norvegicus (Rat) OX=10116 GN=C1r PE=1 SV=1
Ig-like domain-containing protein OS=Rattus norvegicus (Rat) OX=10116 GN=ENSRNOG00000055193 PE=1 SV=
CD5 antigen-like OS=Rattus norvegicus (Rat) OX=10116 GN=Cd5l PE=1 SV=1
HGF activator OS=Rattus norvegicus (Rat) OX=10116 GN=Hgfac PE=1 SV=1
Complement factor H-related protein B OS=Rattus norvegicus (Rat) OX=10116 GN=LOC100361907 PE=1 SV=1
Ig-like domain-containing protein OS=Rattus norvegicus (Rat) OX=10116 GN=ENSRNOG00000048425 PE=1 SV=
Lysyl oxidase-like 1 OS=Rattus norvegicus (Rat) OX=10116 GN=Loxl1 PE=2 SV=1
Coagulation factor X OS=Rattus norvegicus (Rat) OX=10116 GN=F10 PE=2 SV=1
SMB domain-containing protein OS=Rattus norvegicus (Rat) OX=10116 GN=Vtn PE=1 SV=1
Inter alpha-trypsin inhibitor heavy chain 4 OS=Rattus norvegicus (Rat) OX=10116 GN=Itih4 PE=1 SV=1
Immunoglobulin heavy constant mu OS=Rattus norvegicus (Rat) OX=10116 GN=Ighm PE=1 SV=1
Uncharacterized protein OS=Rattus norvegicus (Rat) OX=10116 GN=Cfhr2 PE=1 SV=3
SCY domain-containing protein OS=Rattus norvegicus (Rat) OX=10116 GN=Ccl9 PE=1 SV=1
SERPIN domain-containing protein OS=Rattus norvegicus (Rat) OX=10116 GN=Serpinf1 PE=1 SV=1
Uncharacterized protein OS=Rattus norvegicus (Rat) OX=10116 GN=ENSRNOG00000049829 PE=1 SV=2
Tropomyosin beta chain OS=Rattus norvegicus (Rat) OX=10116 GN=Tpm2 PE=1 SV=1
Apolipoprotein C-II (Predicted) OS=Rattus norvegicus (Rat) OX=10116 GN=Apoc2 PE=1 SV=1
Lipocln_cytosolic_FA-bd_dom domain-containing protein OS=Rattus norvegicus (Rat) OX=10116 GN=C8g PE=1
MACPF domain-containing protein OS=Rattus norvegicus (Rat) OX=10116 GN=C8a PE=1 SV=1
Immunoglobulin joining chain OS=Rattus norvegicus (Rat) OX=10116 GN=Jchain PE=1 SV=1
Inter-alpha-trypsin inhibitor heavy chain 2 OS=Rattus norvegicus (Rat) OX=10116 GN=Itih2 PE=1 SV=3
RNAse_Pc domain-containing protein OS=Rattus norvegicus (Rat) OX=10116 GN=Ang2 PE=1 SV=1
PGRP domain-containing protein OS=Rattus norvegicus (Rat) OX=10116 GN=Pglyrp2 PE=1 SV=2
Complement component factor h-like 1 OS=Rattus norvegicus (Rat) OX=10116 GN=Cfhr1 PE=1 SV=1
Complement factor H OS=Rattus norvegicus (Rat) OX=10116 GN=Cfh PE=1 SV=1
Macrophage-stimulating 1 OS=Rattus norvegicus (Rat) OX=10116 GN=Mst1 PE=3 SV=1
Similar to complement factor H-related protein OS=Rattus norvegicus (Rat) OX=10116 GN=RGD1564614 PE=1
SERPIN domain-containing protein OS=Rattus norvegicus (Rat) OX=10116 GN=Serpinf2 PE=1 SV=1
Ig-like domain-containing protein OS=Rattus norvegicus (Rat) OX=10116 GN=ENSRNOG00000048167 PE=1 SV=
Uncharacterized protein OS=Rattus norvegicus (Rat) OX=10116 GN=ENSRNOG00000042228 PE=1 SV=3
Retinoic acid receptor responder (Tazarotene induced) 2 OS=Rattus norvegicus (Rat) OX=10116 GN=Rarres2 P
SERPIN domain-containing protein OS=Rattus norvegicus (Rat) OX=10116 GN=Serpinc1 PE=1 SV=1
Coagulation factor XIII A chain OS=Rattus norvegicus (Rat) OX=10116 GN=F13a1 PE=2 SV=3
Complement C7 OS=Rattus norvegicus (Rat) OX=10116 GN=C7 PE=1 SV=1
Complement C4 OS=Rattus norvegicus (Rat) OX=10116 GN=C4 PE=1 SV=3
Protein AMBP OS=Rattus norvegicus (Rat) OX=10116 GN=Ambp PE=1 SV=1
Ig-like domain-containing protein OS=Rattus norvegicus (Rat) OX=10116 GN=ENSRNOG00000042996 PE=1 SV=
Ig-like domain-containing protein OS=Rattus norvegicus (Rat) OX=10116 GN=ENSRNOG00000058824 PE=1 SV=

Ig-like domain-containing protein OS=Rattus norvegicus (Rat) OX=10116 GN=ENSRNOG00000042319 PE=1 SV=
Beta-2-microglobulin OS=Rattus norvegicus (Rat) OX=10116 GN=B2m PE=1 SV=1
Ig-like domain-containing protein OS=Rattus norvegicus (Rat) OX=10116 GN=ENSRNOG00000050708 PE=1 SV=
Ig-like domain-containing protein OS=Rattus norvegicus (Rat) OX=10116 GN=ENSRNOG00000047915 PE=4 SV=

=2

=1

. SV=1

SV=1

=3

E=1 SV=1

=2
=2

=1

=1
=1

Accession	Peptide unique T30	Peptide unique TS0	Coverage T30	Coverage TS0	Area CS0 T0 1	Area CS0 T0 3	Area CS0 T0 4	Area CS0 T0 5	Area CS0 T0 6	Area CS0 T0 7
P06399	380	318	85	85	1,76E+09	1,44E+09	6,38E+08	1,39E+09	8,77E+08	1,30E+09
P04937	349	341	78	74	6,85E+08	5,84E+08	3,48E+08	5,20E+08	80200000	1,95E+08
P14480	377	256	94	94	8,12E+08	9,50E+08	3,59E+08	4,95E+08	4,17E+08	5,14E+08
G3V9R2	NA	245	NA	91	5,53E+08	4,77E+08	1,22E+08	1,35E+08	2,50E+08	2,59E+08
P02770	163	226	94	96	1,05E+08	2,59E+09	1,63E+08	57200000	1,44E+09	1,24E+08
Q7TMA5	560	387	74	55	3,50E+08	1,95E+08	79600000	85100000	1,20E+08	70500000
Q01177	168	154	89	86	2,56E+08	1,62E+08	57500000	1,21E+08	1,60E+08	1,47E+08
P02680	261	160	97	94	5,66E+08	5,20E+08	1,96E+08	1,00E+08	1,89E+08	1,27E+08
P02651	210	163	95	95	4,66E+08	5,22E+08	1,77E+08	3,08E+08	1,84E+08	4,68E+08
D3ZFH5	17	32	23	26	3990000	5660000	742000	2370000	3430000	324000
P02650	181	156	94	94	8,96E+08	1,32E+09	4,54E+08	8,41E+08	5,11E+08	9,29E+08
Q63514	118	104	80	77	2,35E+08	3,56E+08	96700000	97600000	1,22E+08	1,46E+08
P04639	219	136	93	93	3,78E+08	5,88E+08	1,82E+08	1,37E+08	1,24E+08	3,51E+08
P14046	183	143	73	70	6290000	1,30E+08	12800000	6330000	53700000	3010000
P13635	167	124	87	76	730000	30600000	2930000	151000	5340000	2820000
P18292	160	107	75	70	1,32E+08	2,32E+08	53600000	81200000	63900000	2,22E+08
Q63041	164	105	83	63	66600000	1,20E+08	25600000	16300000	55800000	9090000
Q03626	179	125	71	62	1120000	22200000	1650000	180000	9210000	NA
P01026	189	145	82	73	14800000	1,12E+08	13200000	6170000	5,30E+07	6620000
P24090	72	58	89	88	3,40E+07	3120000	8910000	2430000	11900000	17700000
P17475	59	71	69	65	3,90E+08	2,63E+08	1,23E+08	3,59E+08	1,35E+08	9,20E+07
P12346	58	73	61	76	3380000	95100000	4930000	9260000	63800000	3050000
B5DEH7	NA	65	NA	75	19500000	23200000	5900000	7610000	6510000	17100000
[illegible]	NA	79	NA	72	19300000	19700000	5300000	893000	16800000	19900000
Q8R2H5	113	64	79	53	46800000	21400000	8410000	11400000	10800000	7700000
P05371	89	69	78	59	1,32E+08	1,16E+08	42500000	65400000	73500000	72600000
D3ZWD6	NA	48	NA	77	7850000	12900000	5280000	1290000	8160000	11100000
F1LPW0	NA	49	NA	60	1,15E+08	1,24E+08	23800000	27200000	42800000	19100000
Q5EBC0	NA	67	NA	74	1,80E+07	30800000	6790000	8050000	12400000	10200000
F1LTN6	NA	34	NA	59	1,80E+07	29900000	6220000	4090000	2210000	2410000
M0R4B5	NA	35	NA	59	44900000	22100000	13400000	18500000	1,10E+07	16400000
P09006	27	41	63	76	29300000	11800000	6150000	25300000	12200000	533000
G3V9J1	NA	69	NA	57	4760000	6950000	2640000	2030000	1690000	7230000
P05544	45	37	80	70	23700000	22400000	5670000	21700000	13900000	1300000
P09005	NA	11	NA	42	6320000	5640000	1990000	6720000	4450000	341000
Q802A3	NA	49	NA	74	24400000	9480000	4810000	6970000	2980000	5160000
G3V8D4	NA	32	NA	80	44800000	1,50E+08	39900000	46500000	64700000	1,66E+08
P53813	51	50	42	44	26800000	23600000	8370000	15600000	17700000	23700000
Q62894	49	43	74	66	25400000	23900000	9060000	2,40E+07	9050000	19600000
Q6P6T1	69	44	62	59	1720000	7020000	1080000	685000	676000	3860000
P55159	81	36	95	79	48600000	56700000	15900000	10100000	11600000	19300000
Q5I0M3	NA	32	NA	65	34300000	30600000	11500000	15600000	1,90E+07	30100000
Q62930	47	39	59	53	19100000	5710000	2510000	7110000	4110000	2630000
P20760	46	34	94	94	13200000	1,26E+08	16200000	33600000	43200000	3710000
P08934	51	44	65	55	10100000	12400000	2990000	11800000	10700000	14300000
P20059	NA	34	NA	63	502000	56400000	7,00E+05	236000	18900000	194000
Q5FWS5	NA	32	NA	59	10500000	8930000	2490000	4750000	6370000	5590000
P14272	52	41	65	55	17200000	6400000	2220000	5220000	4380000	5490000
P04276	32	38	57	71	485000	29700000	1480000	324000	19900000	405000
P55314	48	33	66	55	3750000	2760000	2040000	174000	1100000	2,00E+06
P06866	26	37	63	73	20300000	21300000	2820000	139000	2,60E+07	147000
P19999	31	33	58	62	13500000	3160000	3230000	29400000	5700000	6970000
Q63515	23	18	72	60	13900000	25800000	5550000	11600000	7930000	12300000
P04638	39	14	87	77	8540000	21900000	5110000	7460000	19700000	38700000
P23764	43	27	68	63	25700000	14400000	2430000	1250000	3170000	489000
P19939	45	27	85	77	14400000	5,20E+07	13400000	24400000	19300000	7,40E+07
Q5M7T5	NA	26	NA	54	33400000	7460000	613000	575000	9500000	2260000
D3ZTE0	33	28	49	51	6160000	3450000	1210000	5330000	2760000	3610000
Q7TQ11	NA	24	NA	32	20400000	14800000	3300000	5610000	10900000	11300000
O55004	18	16	73	72	4700000	2690000	1840000	8690000	2070000	6060000
P00924	34	28	57	46	8310000	15800000	3140000	14800000	4540000	8830000
Q63416	22	26	29	36	188000	8560000	72900	27800	4650000	NA
Q9WTS8	28	23	69	55	11100000	7660000	1860000	695000	2930000	1060000
P31720	19	18	60	63	14500000	7810000	2640000	3450000	1900000	2220000
Q99PS8	34	30	50	50	1,40E+07	21300000	3750000	9250000	5840000	517000
P06765	8	14	53	53	2650000	1780000	1580000	161000	355000	126000
Q5FVN3	NA	14	NA	79	797000	NA	NA	22500	173000	267000
Q9QX79	NA	21	NA	56	241000	6640000	NA	NA	2660000	123000
P20759	39	24	83	61	12600000	19500000	8010000	2840000	3420000	1690000
Q68FP1	47	26	59	38	186000	3710000	152000	248000	1120000	142000
P02767	17	16	84	84	6030000	6800000	2460000	3810000	5080000	2570000
P20762	23	21	73	70	28400000	20100000	1140000	622000	7660000	14500000
F1LWS4	NA	25	NA	44	7620000	4930000	1,00E+06	159000	1680000	2120000
Q9EQV8	18	17	47	42	3610000	1330000	588000	1180000	1140000	433000
A0A0G2JYC4	NA	19	NA	48	1790000	1030000	120000	183000	513000	679000
Q63556	NA	16	NA	40	5490000	1830000	840000	3570000	1730000	150000
Q5M8C3	NA	22	NA	56	13400000	2580000	585000	249000	1380000	31400
Q6IRK9	16	17	41	37	2400000	1650000	345000	3200000	1820000	590000
P14630	26	20	64	53	2040000	1800000	475000	498000	493000	486000

Accession	Area CS0 TS0 1	Area CS0 TS0 3	Area CS0 TS0 4	Area CS0 TS0 5	Area CS0 TS0 6	Area CS0 TS0 7	Area LS0 T0 1	Area LS0 T0 10	Area LS0 T0 11	Area LS0 T0 12	Area LS0 T0 13
P06399	1,08E+09	2,16E+09	1,85E+09	2,79E+09	1,39E+09	4,11E+08	2,97E+09	5,96E+09	3,71E+09	3,27E+09	4,49E+09
P04937	5,96E+08	1,07E+09	1,21E+09	7,64E+08	3,85E+08	1,84E+08	1,85E+09	1,61E+09	7,58E+08	8,01E+08	8,41E+08
P14480	5,92E+08	8,19E+08	9,28E+08	9,93E+08	5,14E+08	1,59E+08	1,91E+09	3,77E+09	2,54E+09	1,55E+09	2,65E+09
G3V9R2	2,14E+08	2,52E+08	3,07E+08	4,33E+08	2,58E+08	60600000	4,98E+08	5,09E+08	5,41E+08	4,33E+08	1,06E+09
P02770	1,10E+09	1,07E+09	69900000	1,23E+09	2,28E+08	1,08E+08	2,15E+08	1,75E+08	3,35E+08	1,70E+08	4,32E+08
Q7TMA5	1,11E+08	1,63E+08	2,02E+08	3,11E+08	55600000	27700000	5,03E+08	1,11E+08	5,72E+08	3,28E+08	3,99E+08
Q01177	81500000	1,19E+08	1,24E+08	3,01E+08	99900000	34800000	2,53E+08	87500000	2,82E+08	2,60E+08	5,06E+08
P02680	3,08E+08	4,11E+08	5,17E+08	2,36E+08	1,87E+08	73700000	9,81E+08	2,10E+09	1,43E+09	6,50E+08	1,26E+09
P02651	2,04E+08	6,30E+08	4,47E+08	6,50E+08	2,69E+08	90100000	6,40E+08	1,50E+09	1,34E+09	1,55E+09	1,63E+09
D3ZFH5	3470000	4540000	4970000	2050000	894000	251000	1200000	372000	2890000	2050000	3780000
P02650	4,64E+08	1,47E+09	1,15E+09	1,32E+09	8,35E+08	2,23E+08	9,37E+08	2,94E+09	1,63E+09	1,31E+09	2,49E+09
Q63514	1,17E+08	2,80E+08	2,46E+08	3,28E+08	89400000	44500000	3,63E+08	2,56E+08	6,18E+08	2,90E+08	6,74E+08
P04639	1,98E+08	5,75E+08	3,52E+08	3,75E+08	1,94E+08	91500000	7,70E+08	4,22E+09	1,59E+09	1,63E+09	1,61E+09
P14046	56400000	7890000	12900000	25700000	3980000	2270000	10300000	10800000	43200000	16700000	17500000
P13635	6430000	7800000	6420000	3550000	763000	126000	15500000	7,46E+08	81500000	27500000	32400000
P18292	95700000	1,98E+08	1,88E+08	4,71E+08	2,02E+08	44500000	4,23E+08	5,26E+08	8,81E+08	7,35E+08	7,04E+08
Q63041	80900000	35400000	62800000	36300000	12800000	4250000	37700000	36200000	1,03E+08	56100000	55100000
Q03626	1,00E+07	450000	1700000	3400000	230000	197000	3210000	3290000	11900000	2210000	5300000
P01026	63100000	14700000	1,60E+07	22700000	4320000	1920000	10100000	2,10E+08	3,50E+07	27600000	26100000
P24090	22900000	24200000	24600000	44600000	24700000	5530000	30800000	1,04E+08	30100000	3,50E+07	48300000
P17475	2,39E+08	5,49E+08	4,50E+08	3,74E+08	2,50E+08	86400000	1,99E+08	8540000	1,62E+08	3,25E+08	3,47E+08
P12346	41900000	5740000	3360000	6320000	8700000	3160000	4340000	7190000	9160000	10800000	14400000
B5DEH7	8570000	26600000	14700000	27100000	9040000	2970000	36500000	65200000	71400000	49700000	63800000
[illegible]	6560000	8350000	1,30E+07	35500000	8870000	7750000	5,00E+07	19800000	41800000	2,80E+07	65700000
Q8R2H5	14500000	22100000	29800000	23700000	9600000	2300000	50200000	1,10E+08	1,09E+08	62100000	71600000
P05371	58100000	1,13E+08	90700000	1,66E+08	53900000	1,30E+07	2,68E+08	3,83E+08	4,36E+08	3,14E+08	4,25E+08
D3ZWD6	1240000	11300000	16700000	3380000	7910000	992000	15600000	20700000	19600000	25200000	12500000
F1LPW0	45700000	1,28E+08	61400000	9,90E+07	33100000	5120000	1,42E+08	1,61E+08	1,94E+08	1,37E+08	1,73E+08
Q5EBC0	12900000	18600000	14400000	21200000	7900000	1770000	2,50E+07	4670000	70600000	30500000	57700000
F1LTN6	9470000	15500000	13100000	28300000	15200000	187000	11200000	2710000	27700000	1,20E+07	15600000
M0R4B5	16800000	35700000	32200000	3,90E+07	10800000	4040000	42800000	30800000	76300000	56300000	94500000
P09006	15100000	35700000	43200000	1,20E+07	18200000	2440000	2450000	1740000	4790000	5060000	4960000
G3V9J1	2050000	3710000	3330000	9680000	2060000	2,00E+05	21800000	18200000	30600000	23100000	41700000
P05544	19500000	22900000	27300000	13900000	8120000	2730000	6650000	8180000	6300000	11500000	14800000
P09005	5770000	7650000	7850000	4480000	2620000	1130000	2130000	2330000	1650000	4070000	4940000
Q802A3	6860000	18700000	14900000	13400000	3560000	1450000	15100000	19900000	32300000	29900000	41300000
G3V8D4	37300000	86500000	63700000	2,02E+08	66800000	17700000	2,43E+08	4,79E+08	6,17E+08	3,08E+08	4,63E+08
P53813	10500000	33900000	26100000	48800000	15400000	5850000	38300000	23800000	82700000	64800000	79200000
Q62894	16700000	34900000	25800000	66900000	19800000	4630000	76600000	56500000	32500000	31400000	53300000
Q6P6T1	1030000	3640000	2220000	3600000	969000	139000	26500000	52800000	60700000	19300000	36900000
P55159	22800000	41300000	40300000	44800000	13900000	5570000	85900000	2,47E+08	3,04E+08	1,13E+08	1,93E+08
Q5I0M3	17700000	28300000	26500000	48200000	27800000	5030000	37600000	50800000	62300000	39300000	99400000
Q62930	4110000	7230000	10700000	9030000	4250000	1200000	10600000	5500000	14900000	10400000	16500000
P20760	14600000	78200000	29200000	94600000	23900000	621000	7280000	89100000	5,30E+07	18600000	8260000
P08934	8190000	13800000	7800000	17800000	11500000	2120000	10700000	88200000	18400000	32600000	21900000
P20059	22900000	84000	64300	1,50E+07	860000	342000	363000	283000	3130000	66600	4060000
Q5FWS5	4850000	17700000	9450000	7630000	4240000	978000	19800000	6970000	16800000	25600000	35800000
P14272	3040000	7940000	9580000	10800000	5350000	4020000	6510000	10100000	12200000	8670000	24900000
P04276	10900000	1010000	105000	1,10E+07	1700000	328000	1060000	3910000	2720000	2580000	4210000
P55314	317000	3530000	8810000	350000	2020000	446000	6120000	8730000	9450000	5100000	1970000
P06866	13100000	3860000	92100	442000	948000	180000	7130000	1750000	1970000	7060000	3400000
P19999	4480000	13400000	18800000	13600000	16200000	3910000	24100000	14500000	25200000	12900000	52300000
Q63515	6940000	31200000	15900000	29700000	5990000	2280000	27100000	19400000	48100000	33800000	44400000
P04638	8670000	11100000	9350000	31100000	9300000	3780000	28500000	2,00E+08	8,20E+07	65300000	67100000
P23764	5450000	13400000	9270000	3860000	1370000	674000	21700000	89200000	3,90E+07	14700000	36300000
P19939	17600000	35800000	25500000	71700000	33400000	6260000	64800000	2,18E+08	1,69E+08	1,13E+08	1,63E+08
Q5M7T5	2480000	796000	550000	16400000	1050000	259000	4940000	10700000	2040000	1940000	15500000
D3ZTE0	2230000	5290000	4500000	10700000	5070000	649000	3100000	10400000	8390000	6540000	12900000
Q7TQ11	5740000	9610000	8420000	11300000	6130000	1420000	23200000	30800000	35200000	21700000	36400000
O55004	2280000	10300000	4660000	15400000	6110000	1160000	7520000	5580000	7570000	7350000	12400000
P00924	5330000	12200000	19100000	15700000	1,30E+07	2120000	7240000	5540000	10800000	1,30E+07	14400000
Q63416	3540000	271000	271000	1300000	166000	27700	231000	1270000	1170000	348000	2030000
Q9WTS8	3200000	5820000	4900000	2980000	1120000	139000	13800000	6900000	17500000	8820000	16500000
P31720	2760000	9770000	13300000	7900000	3590000	337000	18400000	1,60E+07	27800000	22200000	24700000
Q99PS8	7880000	1,90E+07	18400000	18300000	2350000	389000	901000	11800000	12300000	1690000	21800000
P06765	265000	4170000	78500	1,04E+08	155000	367000	2240000	696000	116000	6900000	801000
Q5FVN3	294000	621000	116000	73600	377000	NA	358000	717000	NA	302000	752000
Q9QX79	2680000	143000	17700	2890000	64400	26100	221000	30600	263000	251000	265000
P20759	13700000	11500000	1,30E+07	10100000	1430000	213000	1510000	1,70E+07	7320000	12700000	2340000
Q68FP1	896000	881000	454000	490000	NA	5620	457000	10800000	1690000	1510000	1750000
P02767	3450000	7080000	6650000	15800000	3210000	1580000	5080000	1150000	6500000	6640000	8970000
P20762	2300000	1690000	2620000	2510000	3460000	NA	621000	9990000	30900000	1430000	1850000
F1LWS4	1450000	2230000	2310000	2630000	983000	323000	6100000	8930000	9790000	5220000	1,20E+07
Q9EQV8	825000	2940000	2470000	1230000	612000	141000	2130000	4030000	3410000	2020000	4720000
A0A0G2JYC4	209000	542000	275000	942000	348000	30900	977000	1,20E+07	2490000	916000	4300000
Q63556	2350000	2690000	3990000	2180000	1570000	121000	472000	270000	357000	1460000	1080000
Q5M8C3	1710000	1920000	4300000	612000	295000	66500	2540000	1880000	7890000	2250000	4370000
Q6IRK9	639000	7590000	1350000	2940000	791000	292000	5240000	715000	18400000	3790000	6880000
P14630	468000	1470000	1770000	1720000	495000	116000	2730000	27100000	6970000	2400000	5980000

Area L100 T100 6	Area L100 T100 7	Area L100 T100 8	Log2FC RC1 T50	Log2FC RC3 T50	Log2FC RC4 T50	Log2FC RC5 T50	Log2FC RC6 T50	Log2FC RC7 T50	Log2FC RL1 T50	Log2FC RL10 T50	Log2FC RL11 T50	Log2FC RL12 T50	Log2FC RL13 T50	Log2FC RL9 T50	Log2FC RC2 T90	Log2FC RC3 T90	Log2FC RC4 T90	Log2FC RC5 T90	Log2FC RC7 T90	Log2FC RC8 T90	Log2FC RL2 T90	Log2FC RL3 T90	Log2FC RL5 T90	Log2FC RL6T90	Log2FC RL7 T90	Log2FC RL8 T90	Description.y
4,72E+09	2,05E+09	1,51E+09	4,490199577	-4,878035733	-3,817135943	5,0853659	-3,549161779	-0,304226093	1,702532097	-1,856137295	-1,492018857	3,480625841	-0,321071642	-2,345387068	6,167418146	5,005743983	1,182453676	NA	-0,153011619	NA	-1,782103869	2,603109847	-0,051039433	-0,810966176	-2,021883118	0,151007788	Fibrinogen alpha chain OS=Rattus norvegicus (Rat) OX=10116 GN=Fga PE=1 SV=3
7,55E+08	5,25E+08	4,88E+08	4,234944793	-1,659317945	1,894302132	5,54728293	-4,80797557	NA	2,303069068	-3,288244969	-3,012383724	3,240928373	-0,014284226	0,341985747	NA	NA	4,469358459	NA	-0,319068441	NA	-2,018650057	1,011328006	-0,148098639	-1,38332864	-1,467358534	1,908625647	Fibronectin OS=Rattus norvegicus (Rat) OX=10116 GN=Fn1 PE=1 SV=2
3,48E+09	1,22E+09	9,63E+08	3,631855092	-4,050322699	-0,553126414	2,77084046	-2,874469118	0,051115316	2,122619287	-3,732291271	-0,211669904	3,805445332	-0,375509135	-0,787949523	4,17990909	1,614709844	5,184286375	-2,157992778	0,156303231	-1,230076568	-1,457850583	1,311169693	-0,609794354	0,872172536	0,259087221	0,161154792	Fibrinogen beta chain OS=Rattus norvegicus (Rat) OX=10116 GN=Fgb PE=1 SV=4
NA	NA	NA	3,389042291	-4,597269396	-1,221507604	4,426499358	-2,658963082	-0,199308808	0,93128725	-2,866248611	-0,680658153	3,94596016	-0,401740677	-0,421003166	3,91281543	1,639349907	3,001027195	-1,935294311	0,15575767	-1,409875794	-1,655281797	1,723531924	0,386468347	0,103093493	-0,95259064	0,59917636	NA
4,59E+08	4,64E+08	4,59E+08	3,16456324	-4,042342513	0,011227255	2,021490955	-3,754081752	-0,407071189	0,671051477	-2,404390255	-3,57696971	1,719583516	0,064488726	-0,703884599	4,155794673	0,792708146	3,612707491	-3,505528033	0,214940118	-1,646908591	-2,688269032	2,301002256	-0,421973546	-0,108252891	-0,634128558	-0,076102979	Serum albumin OS=Rattus norvegicus (Rat) OX=10116 GN=Alb PE=1 SV=2
8,04E+08	3,79E+08	3,24E+08	3,158429363	-5,624490865	0,043068722	4,239465935	-5,32349539	NA	-0,03641064	-2,886210343	-5,777122721	2,074734798	0,382693351	-2,309217043	4,614827141	-0,769455526	4,219459938	-6,49058535	-0,069708972	NA	-4,391582479	1,307355294	-1,653609848	0,21916852	-0,860220162	-0,690267913	Apolipoprotein B-100 OS=Rattus norvegicus (Rat) OX=10116 GN=Apob PE=1 SV=1
58600000	96600000	96500000	3,138850368	-1,971985624	1,131672633	4,55519857	-2,80708478	-4,484199524	0,150140349	-6,611348763	-6,456531444	-1,103287808	0,625934282	0,627308791	7,460067463	1,295148764	-1,169925001	-4,557660538	-1,239071162	-5,440572591	-3,73978061	-1,845241446	-2,338416218	1,462343214	-4,53196331	-1,836260799	Plasminogen OS=Rattus norvegicus (Rat) OX=10116 GN=Plg PE=2 SV=2
2,54E+09	5,83E+08	4,59E+08	2,268196111	-2,074205263	1,578620974	0,982441629	NA	-4,659176989	0,905416631	-3,747692001	-1,87105849	0,594485275	-0,709744125	NA	9,725689862	2,223288227	1,920519936	NA	-0,835139098	NA	-1,747745433	-0,953740475	-2,357178942	0,839145989	-4,475174355	-1,673189684	Fibrinogen gamma chain OS=Rattus norvegicus (Rat) OX=10116 GN=Fgg PE=1 SV=3
1,99E+09	4,53E+08	5,53E+08	2,092042829	-2,929610672	0,277533976	1,879349903	-3,616889142	-1,785724906	1,41325088	-4,42736437	-1,723290657	1,636309381	0,974909019	0,647185914	5,278460066	1,178337241	0,895891597	-2,387631342	-0,418080519	-3,762500686	-1,297266041	-0,550508444	-0,987509056	0,340737945	-1,982200932	0,754523966	Apolipoprotein A-IV OS=Rattus norvegicus (Rat) OX=10116 GN=Apoa4 PE=1 SV=2
343000	3510000	1810000	-0,201453084	-0,318109756	2,74375476	2,74375476	-1,93986184	-0,368306449	2,343407822	1,082893008	-1,098110085	1,328948523	-0,33824075	1,17362167	3,073147673	3,00904514	NA	-3,953540689	-0,197867824	NA	1,585864467	3,827627317	1,624538013	-1,107365788	-0,342334306	2,218147637	Inter-alpha-trypsin inhibitor heavy chain 2 OS=Rattus norvegicus (Rat) OX=10116 GN=Itih2 PE=1 SV=3
1,35E+09	1,28E+09	1,35E+09	1,96142788	2,277295099	NA	2,497295181	-0,049602086	NA	-2,143000287	-0,594064708	-4,617383978	-4,204652379	-0,68578687	NA	NA	NA	NA	-0,554460412	3,036951136	NA	1,007694392	2,237976317	1,041820176	-5,348229966	-1,824913293	1,831357964	Apolipoprotein E OS=Rattus norvegicus (Rat) OX=10116 GN=Apoe PE=1 SV=2
2,88E+08	6,36E+08	4,07E+08	1,027691734	-0,753610377	0,841527411	2,375890923	1,074444693	-1,804180674	0,188108946	-2,83350294	-2,425133377	-0,541460862	0,268080059	1,166649869	4,600644123	-0,2889962	1,149078217	-1,76176568	-0,196142522	-1,618321098	-1,879286134	0,133678435	-0,731511164	1,300659478	-1,857524518	-0,589211974	C4b-binding protein alpha chain OS=Rattus norvegicus (Rat) OX=10116 GN=C4bpa PE=2 SV=1
1,12E+09	4,70E+08	6,28E+08	0,40275917	-1,79464387	0,477701534	1,158827293	-1,05081379	-2,182322028	1,46456577	-1,544575252	1,035046947	1,559427409	-1,289795474	1,442142729	3,84670359	NA	4,689533764	2,312835449	2,735486665	-3,554290805	1,085989142	2,276331228	-0,823711093	0,207941265	-1,762500686	1,760934242	Apolipoprotein A-I OS=Rattus norvegicus (Rat) OX=10116 GN=Apoa1 PE=1 SV=2
66400000	12500000	2,40E+07	0,289506617	-0,538552036	0,928264246	1,555091971	0,791247255	-3,563290708	-0,472571263	-2,885541275	-1,35430107	-1,622470804	-0,178803153	1,165112368	4,131705679	0,673254889	0,40942857	-0,346513733	-0,095419565	-2,413127909	-0,791413378	0,162553013	-2,848982018	0,803036765	-4,042732985	-0,408265494	Alpha-1-inhibitor 3 OS=Rattus norvegicus (Rat) OX=10116 GN=A1i3 PE=1 SV=1
4,50E+08	26800000	2,40E+07	0,280617525	-1,76121314	1,294620749	1,155097584	-2,124121312	-1,096817453	0,931427247	-0,778746698	-2,328948523	0,526354522	0,209241054	0,332775477	3,666603303	-0,554933705	4,152003093	-1,813834358	-0,170986197	-2,287370873	-1,146486088	0,225881407	-1,157280792	-0,11276793	-0,0118741	-0,514573173	Ceruloplasmin OS=Rattus norvegicus (Rat) OX=10116 GN=Cp PE=1 SV=3
4,34E+08	3,60E+08	5,38E+08	0,14543044	-0,688183221	0,849974556	1,493378951	-0,854020694	-2,578754013	0,408084739	-2,468364743	-1,016425548	-0,129767875	-0,024662054	0,759508163	3,949784689	0,241928773	2,96203215	-0,632154979	-0,502500341	-3,378153975	-0,338686613	0,693130806	-0,456730448	1,027093661	-1,906890596	0,174522713	Prothrombin OS=Rattus norvegicus (Rat) OX=10116 GN=F2 PE=1 SV=1
1,33E+08	36300000	37800000	0,120752159	-0,761840263	0,698637476	1,830392458	-1,257981178	-2,988097911	0,253448357	-1,622794574	-0,984318378	-0,562419932	-0,478047297	0,792999629	4,048711414	0,22881869	2,397592365	-0,368908329	-0,40354186	-5,722060097	-0,489515715	0,126972856	-0,659771088	1,451068146	-1,815498997	0,506959989	Alpha-1-macroglobulin OS=Rattus norvegicus (Rat) OX=10116 GN=A1m PE=1 SV=1
12800000	2220000	4270000	0,021795924	-0,980371193	0,871643074	2,059667045	-1,082893008	-3,355875427	-0,447458977	-4,13289427	-1,815293955	-1,664438654	-0,154154764	1,324350757	5,363992154	1,321928095	-0,902702799	-1,024946357	-0,438370003	-2,76121314	-1,961525852	-1,206450877	-3,852442812	1,550648136	-5,206631927	-1,199208937	Murinoglobulin-1 OS=Rattus norvegicus (Rat) OX=10116 GN=Mug1 PE=2 SV=1
76200000	36700000	24800000	-0,094327383	1,070821337	1,659771088	0,10864126	-0,528176475	-2,071882029	-0,948925815	-0,090512293	-0,251374815	0,950939182	0,590416931	3,581314724	4,102146887	NA	4,996459642	0,787455365	1,455384786	1,145323028	2,706408127	2,276517635	0,235599975	-2,77940086	1,934904972	NA	Complement C3 OS=Rattus norvegicus (Rat) OX=10116 GN=C3 PE=1 SV=3
48600000	13200000	14200000	-0,181112682	-1,300267312	0,951686062	1,628503927	-0,557591529	-3,468885535	-0,622865293	-3,359161001	-1,857555735	-1,679666925	0,187085553	1,213913695	4,793867985	0,716660403	-0,052102983	-0,955825145	0,123148231	-2,49450755	-1,63195998	-1,086761312	-3,581846527	1,257421639	-3,662086927	-0,736965594	Alpha-2-HS-glycoprotein OS=Rattus norvegicus (Rat) OX=10116 GN=Ahsg PE=1 SV=2
21500000	1,75E+08	1,40E+08	-0,200791657	0,873570522	1,797847836	0,516790998	2,263184304	-0,083768358	-0,922572218	-2,722107635	-0,49727576	0,302708799	0,163621259	1,612841533	4,547220407	-0,254542945	2,370837695	0,008562014	-0,106041637	0,081724397	0,654225163	0,887035751	-0,565485126	0,772822516	-0,080170349	1,547033045	Alpha-1-antiproteinase OS=Rattus norvegicus (Rat) OX=10116 GN=Serpina1 PE=1 SV=2
7430000	14600000	12300000	-0,230057083	0,645735923	1,050804371	0,89533119	0,12755005	-2,504472583	0,548203951	-1,740480078	-2,05378041	-1,5087382	-0,238363378	1,759180328	4,544320516	-1,132545804	3,131478566	-0,921780558	-0,184888634	-2,89662226	0,135291366	-0,404126725	-1,601364108	0,368494913	-1,580193257	-0,12225575	Serotransferrin OS=Rattus norvegicus (Rat) OX=10116 GN=Tf PE=1 SV=3
NA	NA	NA	-0,281412935	0,031848866	2,267480311	-0,64261016	-0,77553325	1,070389328	2,510771603	-2,698677109	0,535263032	2,070389328	-0,389274403	1,069998936	6,093735438	1,686692388	4,369862572	-2,906890596	0,124121312	-1,666288848	2,217591435	0,614291166	-0,143364175	0,481622751	-1,387023123	0,851136614	NA
NA	NA	NA	-0,302419936	0,154328146	1,38332864	0,593090382	0,104023065	-2,753878977	1,206900245	-2,624763509	-0,247437551	-0,354374717	0,296675303	1,329613378	4,575555047	0,404390255	1,804407638	-0,283453947	-0,214870302	-2,817455512	-0,550692599	-0,15138905	0,110558375	1,374395515	-1,989538061	0,512061954	NA
1,67E+08	25800000	4,10E+07	-0,455882552	-0,214064062	1,370140961	1,004365193	0,301720976	-1,692741594	-0,264545873	-2,466720879	-1,365168945	-0,381306642	-0,11321061	0,910916456	5,009715155	1,48864354	2,193920483	-1,393266877	-1,510961919	-1,577428828	-0,559427409	-0,543263209	-0,327757174	0,942918905	-2,581015316	1,097610797	Phosphatidylinositol-glycan-specific phospholipase D OS=Rattus norvegicus (Rat) OX=10116 GN=Gpld1 PE=2 SV=1
3,57E+08	1,69E+08	2,08E+08	-0,463131787	0,77842243	1,437736548	0,660250628	-0,37049106	-1,560300446	0,399210016	-1,268629869	-1,004601902	-0,571033662	0,135189128	1,236198221	3,147617999	-0,823122238	4,214124805	0,073373186	-0,259867127	-1,47533801	0,926564553	0,89556259	-0,276587145	0,449741723	0,142994788	1,547220407	Clusterin OS=Rattus norvegicus (Rat) OX=10116 GN=Clu PE=1 SV=2
NA	NA	NA	-0,4639471	-0,228624375	1,810427756	2,536175427	1,660467457	-2,318682435	0,124109087	-0,655214697	-2,041531223	-0,921687598	0,239273048	1,082677023	3,362208999	-0,838759596	3,057844075	-0,31657486	0,385814974	-2,084005975	-0,321255353	0,700439718	-0,395928676	0,415868306	-1,122856748	1,027068243	NA
NA	NA	NA	-0,570187148	-0,366538982	1,465160979	0,876087396	1,053549468	-1,678397975	0,741642081	-3,626619485	0,047151093	0,70107373	-1,424026283	1,347923303	6,204681785	1,963371078	0,885541275	-1,508403406	-0,334068658	-2,661414561	-2,091553938	-1,073248982	-0,68323361	1,68251781	-3,496702496	-0,73289593	NA
NA	NA	NA	-0,604980394	0,546216418	1,50978811	1,478971805	1,129510733	-2,081769556	-0,569973327	-1,715842303	-0,031412953	0,217131905	-0,005423672	2,220144357	3,698491446	-1,123735368	3,137503524	1,466781074	0,885667325	-0,734903128	1,112657801	1,596935142	0,098915026	0,708537186	-0,292781749	1,506606863	NA
NA	NA	NA	-0,610332156	0,830074999	1,711562747	0,508659133	-0,163680787	-2,532982972	1,32436714	0,051591732	0,714931083	-0,321174533	0,24423681	1,597476254	3,376693898	-1,325485914	4,371943633	1,27450719	0,310481568	-2,341595128	2,681560124	3,799348269	0,220671033	1,028408415	0,464177789	1,604071324	NA
NA	NA	NA	-0,631912916	-2,464180678	-4,936350196	4,990889582	-4,777480754	0,292180751	-2,547020929	-3,830624701	-0,963840337	1,16218447	-0,229481846	-6,560198261	4,392723873	NA	1,862496476	NA	0,587891832	NA	-2,908852112	0,271302022	-2,088437965	0,900920226	0,746175071	-0,188512463	NA
2270000	7650000	5170000	-0,640712944	-0,373043411	2,604736174	0,085167383	1,517747421	-2,058349173	0,550002662	-0,039600703	-0,140177658	-0,215012891	0,67556505	0,358047521	2,966596842	0,748352354	3,780293026	0,25061069	-1,544661136	-3,835505962	0,627190736	1,268378627	-0,331514144	0,754624261	-0,60334103	1,025355574	Serine protease inhibitor A3N OS=Rattus norvegicus (Rat) OX=10116 GN=Serpina3n PE=1 SV=3
NA	NA	NA	-0,660911354	-0,041820176	1,656169451	0,727976811	0,569855608	-3,579085934	-0,214608303	-4,285402219	-1,995567754	-1,863599033	-0,5360529	3,04940224	4,252980741	-0,358047521	-0,555571651	-1,792903766	-1,392784692	-2,730208228	-1,879358693	-1,496352483	-2,918265385	1,061986887	-2,541606132	-1,378773955	NA
2170000	1950000	1840000	-0,683468046	0,735267307	1,437087293	0,816778176	-0,454565863	-1,675514258	0,415037499	-1,273367737	-1,176156955	-0,672296167	0,137503524	0,952322025	3,584257199	-0,844545481	3,867837819	-0,22650853	-0,101003319	-1,574694165	0,358655029	0,633572445	-0,944022201	0,300467732	-0,099064128	1,169925001	Serine protease inhibitor A3L OS=Rattus norvegicus (Rat) OX=10116 GN=Serpina3l PE=1 SV=3
NA	NA	NA	-0,684290428	1,989352756	1,763299742	-0,921997488	-0,539458677	-1,048782373	1,777764317	2,492943157	1,264514852	0,99765606	-1,881944238	0,478789921	NA	NA	NA	2,273335327	-0,061400545	-2,550840616	4,413469549	6,118686774	6,218249449	NA	3,289772857	NA	NA
NA	NA	NA	-0,703282468	-0,5887205	0,879769253	1,359139174	-0,770201924	-1,521402511	0,408984829	-2,344919738	-1,67844913	-0,676324243	-0,478802042	0,852082286	3,536371763	0,037089319	2,804675822	-1,097663259	-0,601450624	-4,176306853	-0,305855688	0,620476787	-0,86890101	0,368539037	-1,681202323	0,334000927	NA
NA	NA	NA	-0,704544116	0,584962501	1,535896942	1,005180239	0,664436135	-1,661301324	0,125530882	-2,079617168	-0,634408569	-0,434283588	-0,214381879	1,012072832	4,871485259	0,845277405	2,012515951	-1,030338163	-1,0622283	-1,632057618	-0,398698762	-0,064488726	-0,103622631	0,786241935	-2,328948523	0,889676585	NA
22100000	48900000	40500000	-0,706463506	1,06174335	1,871266686	0,059054426	0,888968688	-0,090602549	1,177156571	3,78840698	0,548893246	0,435349955	-0,383509417	1,164386818	-0,063658637	-2,731938777	8,158429363	1,267347399	1,33266337	-1,458249566	3,501906761	5,659797734	3,728184709	-1,006725863	4,004954882	2,975477681	Vitamin K-dependent protein S OS=Rattus norvegicus (Rat) OX=10116 GN=Pros1 PE=2 SV=1
16400000	3,20E+07	25800000	-0,724026538	-1,396566896	0,90231303	2,058283541	-0,72935241	-4,100512778	-0,120512807	-3,934904972	-2,319316881	-0,55480053	0,224215157	0,819427754	5,795859283	0,449766529	3,038395379	-1,901697926	-0,405514725	-2,495893826	-2,390353236	-1,509931791	-3,227493293	0,94058179	-3,309123025	-2,08292096	Extracellular matrix protein 1 OS=Rattus norvegicus (Rat) OX=10116 GN=Ecm1 PE=1 SV=2
96900000	2,70E+07	19100000	-0,739764228	-0,94753258	1,039528364	2,393821013	0,519473419	-4,795444059	-0,718931671	-1,989111684	-4,846381612	-2,24195979	0,741649673	1,062434047	4,836501268	-0,569219764	2,892602209	-2,131406189	0,092691898	-3,061959992	-1,160209847	0,082677023	-1,443129806	-0,01346226	-2,557995453	-0,223471072	Complement C1s subcomponent OS=Rattus norvegicus (Rat) OX=10116 GN=C1s PE=2 SV=2
2,24E+08	57200000	80800000	-0,773584185	-0,043589269	1,888209175	4,245548329	-1,482848283	NA	0,015106892	-2,242360838	-4,832617833	-2,927649156	0,169925001	0,329965414	4,655171503	-1,134868747	0,816242725	-3,178854313	-0,143491594	NA	-1,557641895	-0,360402243	-2,145299553	1,6208077	-3,390969739	-1,150025444	Serum paraoxonase/arylesterase 1 OS=Rattus norvegicus (Rat) OX=10116 GN=Pon1 PE=1 SV=3
NA	NA	NA	-0,80556164	0,058214614	1,434696025	2,052061656	-0,6622552	-0,701843801	0,727277303	-0,064130337	-0,675410924	2,096635783	-0,159707997	1,775828417	4,202660494	1,016775713	5,508987443	-0,078588735	-0,033770737	-3,475668329	1,346802764	1,819427754	0,18895423	-0,520571913	-1,43248647	0,757357383	NA
9720000	18400000	7370000	-0,829115441	1,810729649	2,177962321	3,395928676	0	NA	1,343954401	1,901662081	0,090543092	-1,696486525	-0,341902795	NA	3,574345393	NA	NA	2,478653599	-1,359460151	NA	2,59400764	NA	2,559664467	-0,445898463	-0,288721049	4,59580406	Complement component C9 OS=Rattus norvegicus (Rat) OX=10116 GN=C9 PE=1 SV=1
63300000	61600000	82500000	-0,829159292	-0,164854012	2,294743266	0,984318378	-1,313307612	-0,410394125	3,344172732	-3,463104158	-1,222783449	3,178436501	0,479743189	-0,816288047	2,082868267	-0,313660479	4,920373825	1,575773329	-0,967135366	-2,733192431	2,607330314	3,896906507	1,377701348	-1,632142769	1,532179161	2,389526173	Ig gamma-2A chain C region OS=Rattus norvegicus (Rat) OX=10116 GN=Igg-2a PE=1 SV=1
2,70E+07	17300000	16400000	-0,8397037	-0,299279356	1,406182701	0,721381699	0,687477411	-0,638231641	0,193910045	-0,756190161	-0,845899966	0,513500138	0,327495422	-1,316821204	2,645817706	-0,025952696	2,88430731	-0,503077534	-0,456176194	-1,700007838	0,289128602	1,848882267	-0,385969457	1,330417335	-1,428383525	1,038819249	Kininogen-1 OS=Rattus norvegicus (Rat) OX=10116 GN=Kng1 PE=2 SV=1
NA	NA	NA	-0,877871702	-0,339373229	1,399310626	1,23878686	-0,015347964	-0,785091973	-0,125932021	-2,764710585	-2,187178683	-0,305376919	-0,094636874	0,389770741	5,361172412	1,668218004	2,077275713	-2,217543022	-1,268488836	-1,814548535	-1,242047318	-0,815575429	-0,59269815	0,742634344	-2,71200485	0,781654412	NA
NA	NA	NA	-0,89503044	-0,580774704	1,391830108	0,834765521	0,264177587	-0,23752813	0,639410285	-0,916267733	-0,827684451	-0,163190167	0,12717124	-1,484481108	1,300448367	-1,516345889	2,565561664	-0,71830175	-0,58114079	-1,832055363	0,030214613	2,272600383	-0,932221886	1,634312417	-2,26489715	1,431992977	NA
8990000	6600000	6710000	-0,932885804	-0,032254199	0,951636978	1,452714702	0,645716532	-1,939627382	0,055141554	-3,480784859	-2,286082896	-1,531104897	0,136688672	0,489038081	5,436606699	2,326549653	0,239187664	-1,015824967	-0,521952703	-2,404390255	-0,409390936	-0,843030654	-3,129283017	2,382469637	-4,535523568	-0,047453919	Plasma kallikrein OS=Rattus norvegicus (Rat) OX=10116 GN=Klkb1 PE=1 SV=1
1,30E+07	4580000	4830000	-0,949373927	0,155278225	1,340869659	0,650360224	0,708452906	-2,058634886	-0,072623616	-1,133583154	-0,994699216	-1,138605238	-0,059135124	0,87173415	3,371759251	-0,629859039	1,802235951	-0,038079206	0,188152097	-2,18577383	0,442222329	-0,178597303	-1,167494175	1,964843555	-0,960001932	1,004280997	Vitamin D-binding protein OS=Rattus norvegicus (Rat) OX=10116 GN=Gc PE=1 SV=3
1230000	2370000	4500000	-0,956352115	1,597137215	2,812372997	-1,076102979	0,577057303	2,19467371	3,186190056	0,601450624	1,238225212	2,08712737	-0,321201113	0,993063953	5,147597914	-1,087462841	NA	-0,736965594	0,941290693	-0,789782293	2,439960112	0,26386617	0,208502034	0,292392585	0,362570079	1,79485195	Complement component C8 beta chain OS=Rattus norvegicus (Rat) OX=10116 GN=C8b PE=2 SV=2
3390000	6810000	4370000	-1,002077315	0,274174963	1,518467089	1,356338126	-0,404764863	-2,431552586	0,239779054	-0,669175505	-0,521875798	-0,749827745	-0,490635118	1,148405348	2,852442812	-2,734781347	3,605866604	0,068171503	0,288699289	-3,27100416	0,037614292	0,851901361	-0,291889269	0,563900885	-0,190822342	0,882643049	Haptoglobin OS=Rattus norvegicus (Rat) OX=10116 GN=Hp PE=1 SV=3
9030000	8980000	6950000	-1,006152227	-0,346450414	1,347070521	1,748742762	-0,448534411	-1,714091128	0,375757512	-0,733213459	-0,832671175	-0,163100802	-0,573237541	1,232660757	2,966988817	-2,420525125	3,169305952	-0,267621675	0,184542098	-2,982331352	-0,689659879	0,868868085	-0,610517259	0,588298213	-0,253171083	0,430480245	Mannose-binding protein A OS=Rattus norvegicus (Rat) OX=10116 GN=Mbl1 PE=1 SV=1
18400000	49500000	35400000	-1,043626932	1,93696626	1,340624189	0,825502269	1,561541612	-2,385192988	2,061959992	0,88430731	1,505971454	1,038732394	0,584962501	2,912604247	3,42779673	-1,261627585	5,474518485	1,826720247	0,475753924	-1,179882126	2,321928095	2,038395379	-0,231325546	0,499216132	1,327757174	1,285076738	C4b-binding protein beta chain OS=Rattus norvegicus (Rat) OX=10116 GN=C4bpb PE=2 SV=1
1,56E+08	16600000	23300000	-1,091922489	-0,457206954	1,341753073	2,149143439	0,260960078	-1,792851615	-0,330772553	-1,77844223	-3,969942766	-1,591360272	0,355910194	0,365284464	4,790960484	-0,327164743	1,980891177	-2,580530254	-0,376803281	-3,150505745	-1,864077512	-1,175442006	-2,492728784	0,505528033	-2,428843299	-0,921104455	Apolipoprotein A-II OS=Rattus norvegicus (Rat) OX=10116 GN=Apoa2 PE=2 SV=1
1,02E+08	9810000	11800000	-1,182203331	0,971367847	1,46918127	0,287162677	0,074424841	-1,963667787	1,300659478	-0,275634443	0,664961823	0,012174714	-0,966357156	1,431900937	2,172784656	NA	4,26589406	2,509775004	0,110506791	-2,441152103	2,154373998	0,750828248	0,841496178	0,62943713	-0,038474148	1,294212717	Glutathione peroxidase 3 OS=Rattus norvegicus (Rat) OX=10116 GN=Gpx3 PE=2 SV=2
2,39E+08	39500000	69400000	-1,183927861	-0,037802033	1,09363971	1,343820701	-0,447458977	-2,481457925	-0,33137957	-1,154935827	-2,331889205	-1,424256029	0,169547777	0,762002145	3,814526577	-0,346215467	2,325853798	-1,313104245	0,232429944	-3,004038594	-0,430634354	0,581015316	-0,783437897	0,705095079	-2,096972401	0,049388027	Apolipoprotein C-I OS=Rattus norvegicus (Rat) OX=10116 GN=Apoc1 PE=3 SV=1
NA	NA	NA	-1,191760803	0,271302022	1,336525471	1,077509367	0,547900406	-2,376909519	-0,041157325	-2,011587974	-1,487150477	-1,319603042	-0,348728154	0,947848718	3,976331094	-0,176077228	2,002138911	-0,972554118	-0,652777885	-2,405377388	-1,041820176	1	-1,871920186	-0,05166212	-2,612303021	-1,22201968	NA
3550000	8180000	7770000	-1,351843673	0,522498414	1,640750279	1,645335119	-0,200819009	-2,018378529	0,666726932	-0,317388935	-1,511834429	-0,94753258	-0,0863248	0,682809824	2,535807007	-1,204947287	3,927749201	-0,230612928	-0,095731465	-2,796171184	0,288676289	0,624221931	-0,662615056	0,411769785	-0,754559654	0,622859108	Coagulation factor XII OS=Rattus norvegicus (Rat) OX=10116 GN=F12 PE=2 SV=1
NA	NA	NA	-1,465886641	0,61667136	1,894917954	1,005403359	0,87731748	-2,475708454	1,685325289	-1,567040593	-0,434077542	0,723668772	-0,767101312	1,105891169	2,934904972	-1,05270705	3,890878278	0,729500144	-0,076828304	-4,859695428	1,324330585	1,485426827	-0,298658316	1,358047521	-0,645971062	1,737584644	NA
5950000	12400000	8310000	-1,584962501	0,502500341	2,426036498	1,502500341	-1,136006693	-3,426685836	1,702614089	1,759260634	1,780760712	0,359895945	0,622995633	2,636361653	6,125023278	0,284775096	4,543055549	0,386278506	1,155119067	-1,77909107	2,403392196	3,810564479	1,255526869	-1,328520772	0,029328665	3,317788365	Ribonuclease 4 OS=Rattus norvegicus (Rat) OX=10116 GN=Rnase4 PE=1 SV=1
8120000	10400000	5740000	-1,59138877	2,084236537	2,541126592	-1,112209504	1,506959989	-0,833990049	1,729162357	-1,246808615	0,760812336	0,977457431	-0,964577946	1,15090557	5,117212648	1,486558797	3,612829294	-1,547270211	-1,277770309	-2,419693862	1,109411217	0,050626073	0,473171675	1,585494959	-1,539262457	0,86344034	Enolase 1 OS=Saccharomyces cerevisiae (strain ATCC 204508 / S288c) OX=559292 GN=ENO1 PE=1 SV=3
4800000	1320000	1040000	-1,656795245	-0,25860216	1,343514957	1,869683543	-1,109877618	-1,347737281	-0,43138277	0,088227183	-3,070288444	-2,026635127	-0,350226089	0,48133987	0,746243408	-6,381137783	5,007292493	-1,115317988	1,196588666	-2,95550054	0,144908305	-0,372554168	-0,287504264	-1,601203142	3,499964848	0,432959407	Inter-alpha-trypsin inhibitor heavy chain H3 OS=Rattus norvegicus (Rat) OX=10116 GN=Itih3 PE=2 SV=1
26300000	7050000	5110000	-1,69045563	0,046435573	1,825134625	1,055853235	-0,169925001	-1,743224585	-0,247029701	-2,053439259	-5,117419766	-1,530256609	0,358534566	4,478518458	5,413658366	0,40545145	1,810296197	-2,528077437	-0,715420064	-4,223092079	-1,483471956	-1,128897733	-2,607283547	1,071622928	-2,720091776	-1,026152288	Ficolin-1 OS=Rattus norvegicus (Rat) OX=10116 GN=Fcn1 PE=2 SV=2
23500000	12900000	10200000	-1,781359714	-0,12659325	2,261847491	NA	NA	NA	-0,358686645	-2,405141463	-6,790224227	-2,350098768	-0,088355874	NA	5,735676898	-0,549687026	1,903784685	NA	-0,611331029	NA	-2,900133229	0,180670693	-2,206450877	1,427281095	-2,399683252	-2,594270243	Complement C1q subcomponent subunit A OS=Rattus norvegicus (Rat) OX=10116 GN=C1qa PE=1 SV=2
11500000	21200000	14200000	-1,794415866	-0,396325239	1,397479128	2,100227448	-1,387401932	-2,930907477	-0,108524457	-1,05541312	-2,511044361	-1,003275132	0,034557222	0,570315725	3,898328582	0,281570357	4,720159557	-1,601932137	-0,700318692	-3,792070194	-1,718931671	0	-1,023846742	-0,005495988	-0,926537838	1,442590629	Histidine-rich glycoprotein OS=Rattus norvegicus (Rat) OX=10116 GN=Hrg PE=1 SV=1
4260000	5460000	4300000	-1,816582762	-0,78209497	2,24894865	3,391562283	0,370096606	-3,199071347	0,519751088	-0,7589919	-0,777148633	-0,493498738	-0,415750117	2,646848299	5,775163636	-1,544996093	3,914158596	0,061927749	1,355094959	-4,040641984	-1,91020333	0,090049605	0,559427409	0,203872333	-0,549967079	1,401098308	Platelet factor 4 OS=Rattus norvegicus (Rat) OX=10116 GN=Pf4 PE=1 SV=1
NA	NA	NA	-1,90914657	2,201633861	1,96829114	-0,12225575	-1,202188851	-1,014746585	0,195395689	0,959069736	-3	-1,745875076	-2,05994254	0,528928466	6,036173613	2,497437941	6,070179497	-2,402346777	-0,202273779	NA	1,575879396	1,059609489	2,301002256	3,341749536	-0,781999348	0,797579	NA
NA	NA	NA	-2,123988717	-0,292180751	1,897749942	1,788190918	0,005840879	-2,066831508	-0,228570996	-4,370654135	-2,888892028	-1,514573173	-0,212774419	0,120752159	5,903791357	0,065588342	0,227410496	-2,738428032	-0,600392541	-4,052579915	-2,935869663	-1,460226273	-4,826059521	1,762358752	-4,673218249	-2,259335617	NA
17300000	42900000	3780000	-2,129532813	1,144389909	2,070622982	0,059871456	-0,897430266	-1,618671862	1,248283954	-1,270931736	-2,171406534	-0,088355874	-0,574304019	0,149962506	5,260151897	-0,84064424	4,032421478	-1,727787665	-1,790845835	-4,023618214	1,154123145	0,829531713	-0,948975997	2,071317405	-1,31937239	2,106611191	Ig gamma-1 chain C region OS=Rattus norvegicus (Rat) OX=10116 GN=ENSRNOG00000030332 PE=1 SV=1
10900000	580000	1110000	-2,216362339	0,340504902	2,091851527	0,344876428	0,048324447	-1,132028394	1,130060541	-0,078148246	-0,95238198	-0,028014376	-0,366322214	1,133711945	3,226893814	0,887941493	4,824428435	-0,113239476	0,219889914	-2,700253816	1,530712875	-0,183446141	0,528819423	-0,248855471	-0,610864364	1,163577035	Gelsolin OS=Rattus norvegicus (Rat) OX=10116 GN=Gsn PE=1 SV=1
4820000	2260000	4750000	-2,237440224	-0,103835811	1,931613025	1,626672753	-1,210306947	0,462914126	0,096429763	-3,552972386	-4,759333407	-1,614709844	-0,209204989	-1,450847575	6,818251895	1,452109086	1,584962501	-5,289747689	-0,598312054	-5,514573173	-3,443583887	-1,486738965	-2,048625354	1,541893779	-3,406186582	-1,885541275	Transthyretin OS=Rattus norvegicus (Rat) OX=10116 GN=Ttr PE=1 SV=1
1,80E+07	323000	2410000	-2,393312728	0,323036014	2,332816411	1,195256291	0,917984425	-2,71973918	-0,174833802	-1,936497058	-2,181125904	-1,417639294	0,50284388	0,291181735	4,694630757	-0,15565087	3,842318594	-1,530514717	-0,271027197	-1,811512548	-0,250406798	-0,855051664	-2,532712272	0,25276607	-1,337500899	-0,603699063	Ig gamma-2C chain C region OS=Rattus norvegicus (Rat) OX=10116 GN=- PE=2 SV=1
NA	NA	NA	-2,500265336	0,311067102	2,109465979	1,0489096	0,288608022	-0,449610648	1,232802204	-1,31836148	-1,4911142	-0,236326227	-1,273501405	0,444955022	1,01337903	-3,275252634	4,016425548	-0,937264245	-0,12407435	0,474616391	-0,270397794	0,247469103	-1,957653709	1,475535074	-1,63508598	-1,008574736	NA
1480000	1130000	1460000	-3,321928095	1,228170142	-4,331088094	9,335307125	-1,195550809	1,542356329	2,044394119	1,028731719	5,205414086	-2,046748259	4,473931188	3,309400997	4,039689478	-1,812153091	6,195816768	5,107423583	2,758548744	-5,455945443	4,766230189	1,230497471	-2,897497461	-0,605426603	-1,305986551	-1,480625841	Carboxypeptidase N catalytic chain OS=Rattus norvegicus (Rat) OX=10116 GN=Cpn1 PE=2 SV=1
NA	NA	NA	-3,56433585	0,354999916	2,110572867	1,008267616	0,876851769	-2,164884385	1,030324537	-1,850974606	-2,337276059	0,58118581	-0,4723047	-0,291134941	3,024946357	-0,599110763	4,761609721	-2,562419932	-0,786404527	NA	-0,349584438	-0,62544157	-1,593277769	-0,047696742	-1,507861532	0,22881869	NA
NA	NA	NA	-3,626185163	-3,57210035	1,200532987	2,012700879	-1,146572354	NA	0,850833559	1,953776983	-0,168175219	-0,625373903	-0,015681622	0,187085553	6,569309236	1,53419389	4,376465379	-2,975277571	-0,984893108	NA	-2,561115759	-0,625604485	0,629965995	0,055041364	-3,416990292	0,320432299	NA
NA	NA	NA	NA	0,386124807	2,119266307	NA	NA	NA	-0,33219643	-1,873207469	-5,114236723	-2,702351839	0,426092688	1,763765654	6,565144214	-1,283998639	3,970680608	-3,915018476	0,068947354	NA	-2,031478231	0,633872101	-1,441269041	0,380272081	-2,745329067	-0,678071905	NA
506000	1640000	478000	NA	-0,85170768	NA	NA	-0,147557188	-4,527851864	-0,700439718	-2,971430848	-4,063360241	-2,81788559	-0,215638328	NA	4,785875195	-2,192201922	0,04662616	-2,141495436	-0,097297201	NA	-1,53951953	-0,065376009	-3,902628569	0,738838008	-3,146841388	-2,001820436	Carboxypeptidase Q OS=Rattus norvegicus (Rat) OX=10116 GN=Cpq PE=1 SV=1
21400000	1070000	1790000	NA	3,044049842	NA	0,785149128	0,091922489	NA	-0,235577443	-1,564041849	-4,669747723	-2,99262047	-0,562681053	NA	3,918863237	-2,008173931	5,180373951	-1,916833399	0,304854582	NA	-0,774579886	3,46712601	0,87848218	1,05246742	-2,291894928	0,974389356	Apolipoprotein M OS=Rattus norvegicus (Rat) OX=10116 GN=Apom PE=1 SV=2
